# Supplementary figures and images for: Autophagosome membrane expansion is mediated by the N-terminus and cis-membrane association of human ATG8s
Source: eLife. 2023 Jun 8;12:e89185. doi: 10.7554/eLife.89185 (PMC10289813; doi:10.7554/eLife.89185)

C

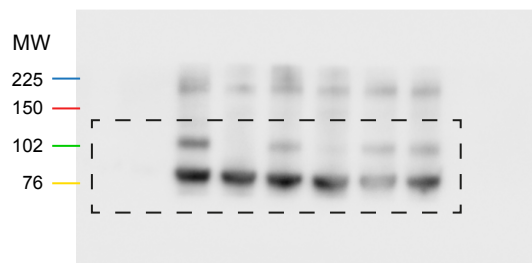

ATG7

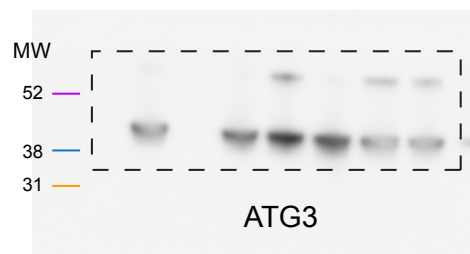

ATG3

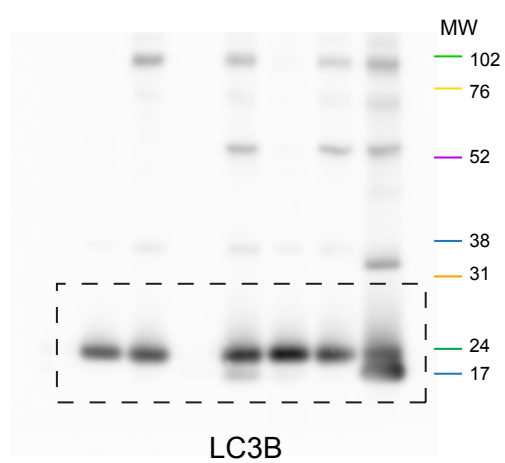

LC3B

E

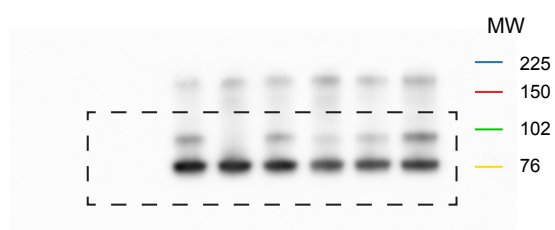

ATG7

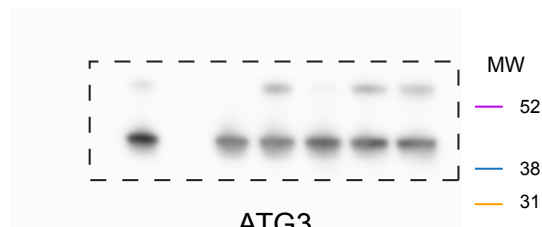

ATG3

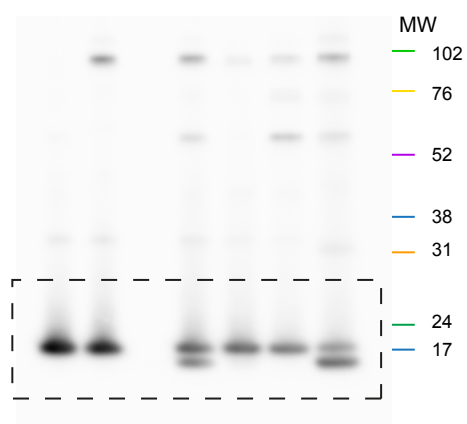

GABARAP

Figure 1

Supplement: Figure 1—source data 1. [file elife-89185-fig1-data1.zip › Figure 1-source data 1/Figure 1-source data_labelled.pdf]

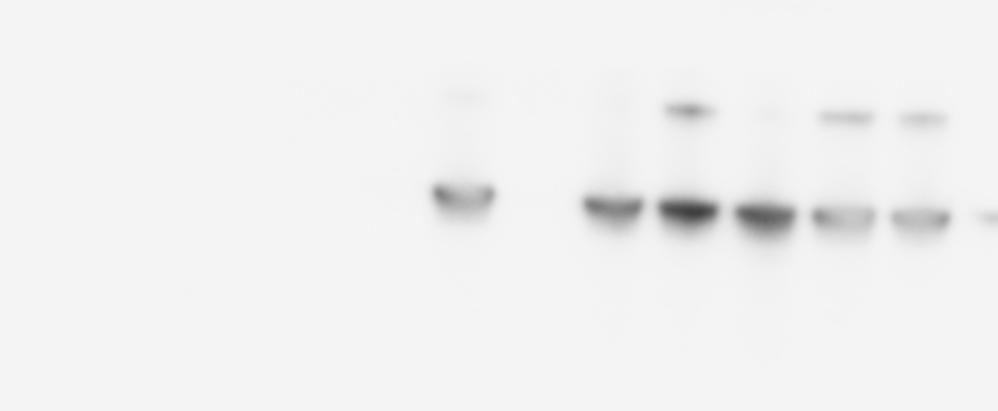

Supplement: Figure 1—source data 1. [file elife-89185-fig1-data1.zip › Figure 1-source data 1/Fig. 1C/2021.02.17_11.23.24_Ch_ATG3.tif]

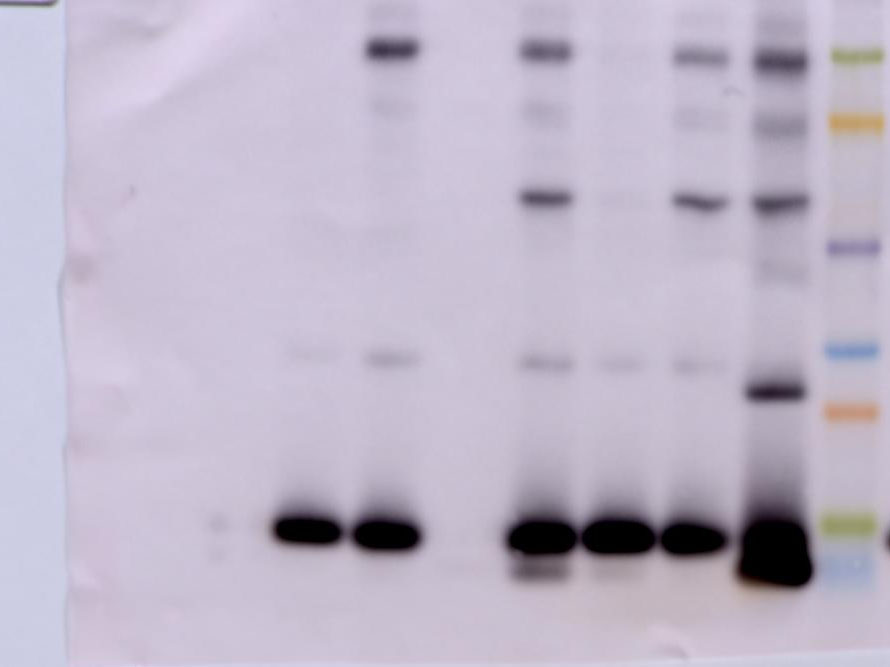

Supplement: Figure 1—source data 1. [file elife-89185-fig1-data1.zip › Figure 1-source data 1/Fig. 1C/2021.02.17_11.23.24_Ch+Marker_LC3B.jpg]

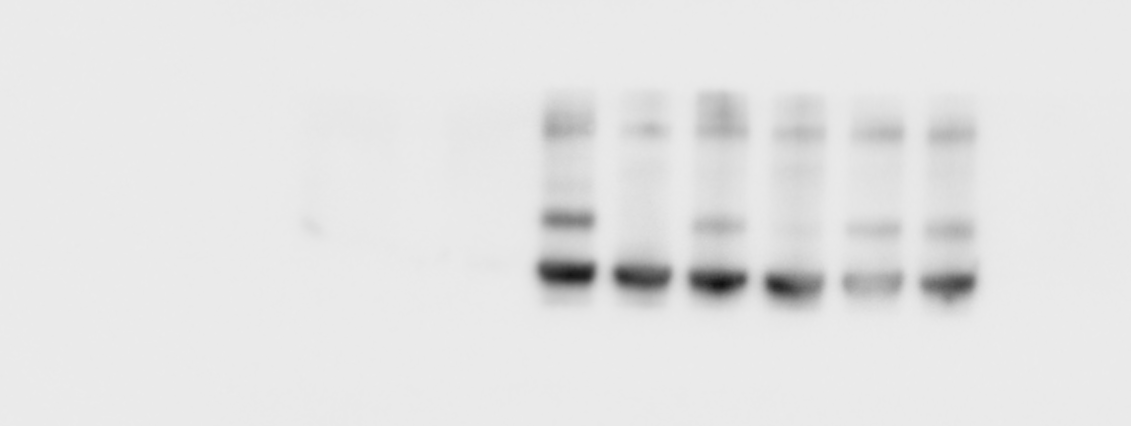

Supplement: Figure 1—source data 1. [file elife-89185-fig1-data1.zip › Figure 1-source data 1/Fig. 1C/2021.02.17_11.23.24_Ch_ATG7.tif]

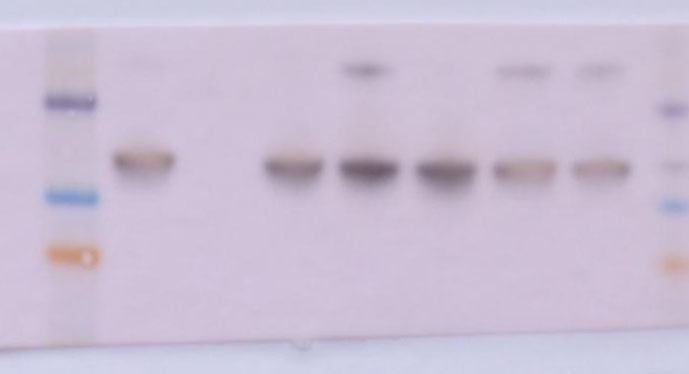

Supplement: Figure 1—source data 1. [file elife-89185-fig1-data1.zip › Figure 1-source data 1/Fig. 1C/2021.02.17_11.23.24_Ch+Marker_ATG3.jpg]

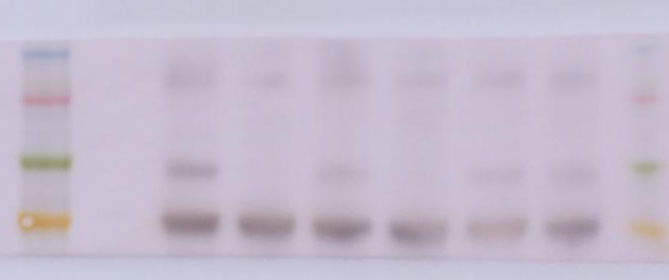

Supplement: Figure 1—source data 1. [file elife-89185-fig1-data1.zip › Figure 1-source data 1/Fig. 1C/2021.02.17_11.23.24_Ch+Marker_ATG7.jpg]

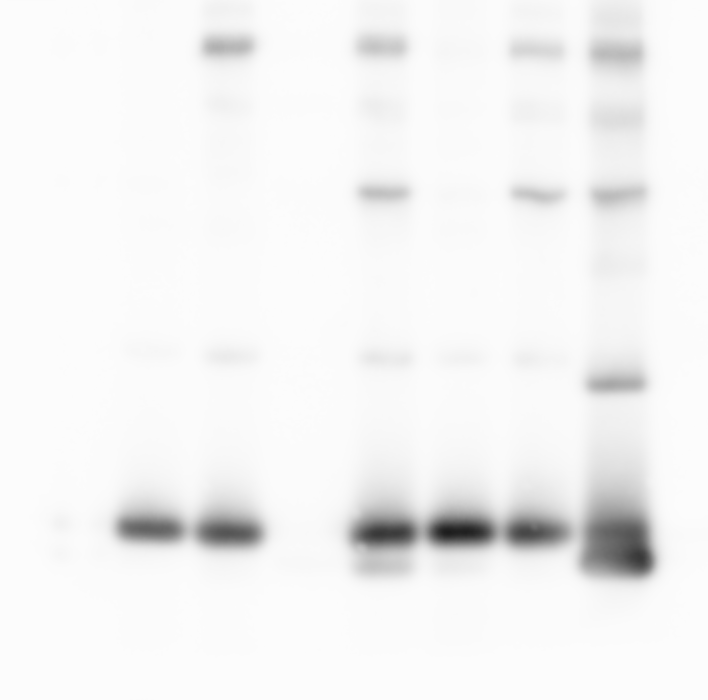

Supplement: Figure 1—source data 1. [file elife-89185-fig1-data1.zip › Figure 1-source data 1/Fig. 1C/2021.02.17_11.23.24_Ch_LC3B.tif]

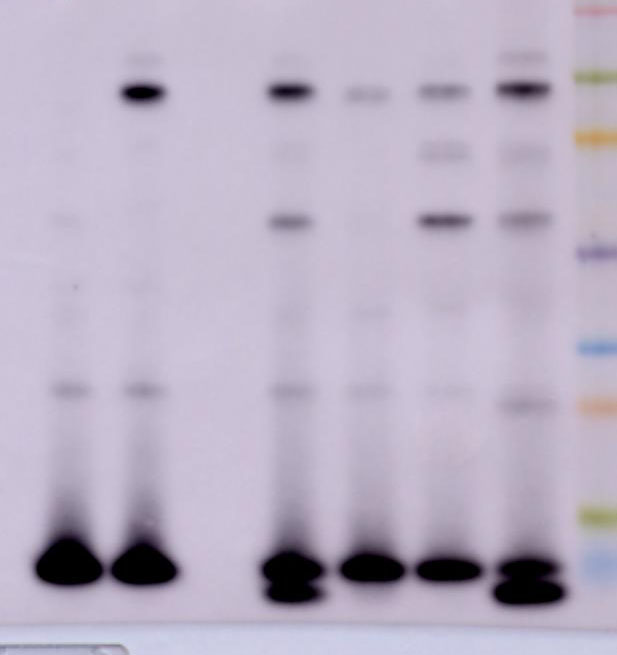

Supplement: Figure 1—source data 1. [file elife-89185-fig1-data1.zip › Figure 1-source data 1/Fig. 1E/2021.05.05_07.46.07-08_Ch+Marker_GAB.jpg]

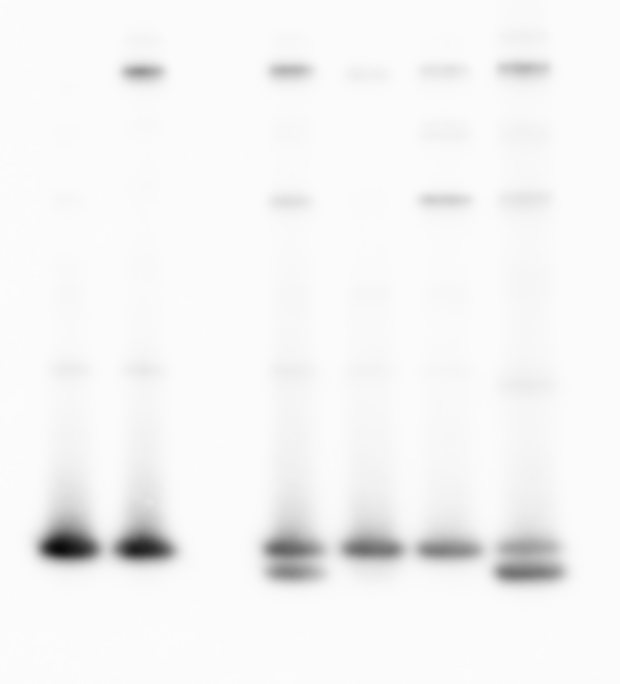

Supplement: Figure 1—source data 1. [file elife-89185-fig1-data1.zip › Figure 1-source data 1/Fig. 1E/2021.05.05_07.46.07-08_Ch_GAB.tif]

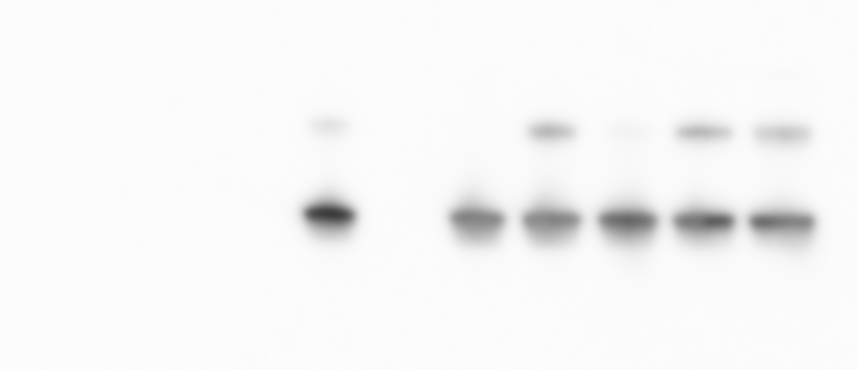

Supplement: Figure 1—source data 1. [file elife-89185-fig1-data1.zip › Figure 1-source data 1/Fig. 1E/2021.05.05_07.59.06-12_Ch_ATG3.tif]

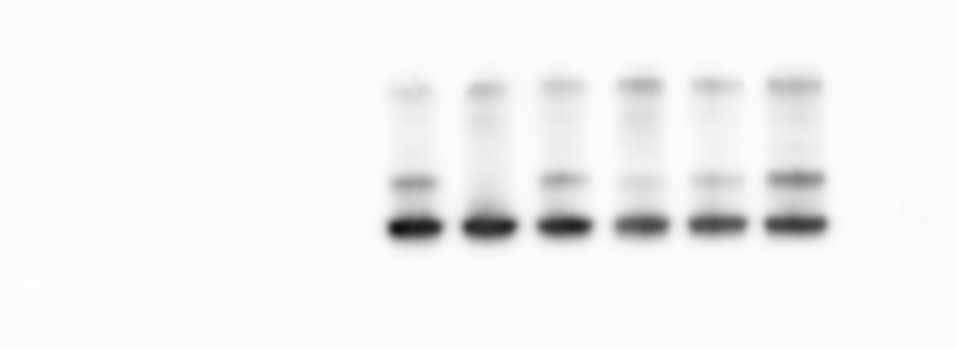

Supplement: Figure 1—source data 1. [file elife-89185-fig1-data1.zip › Figure 1-source data 1/Fig. 1E/2021.05.05_07.59.06-12_Ch_ATG7.tif]

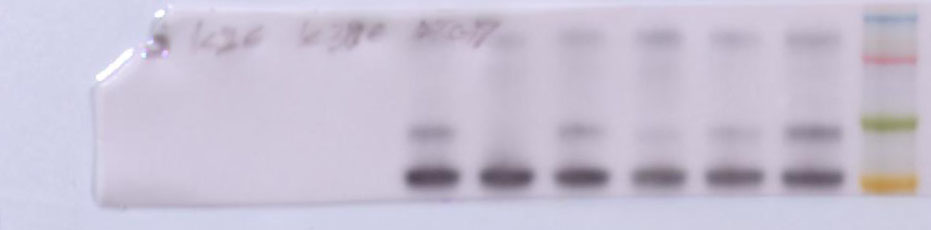

Supplement: Figure 1—source data 1. [file elife-89185-fig1-data1.zip › Figure 1-source data 1/Fig. 1E/2021.05.05_07.59.06-12_Ch+Marker_ATG7.jpg]

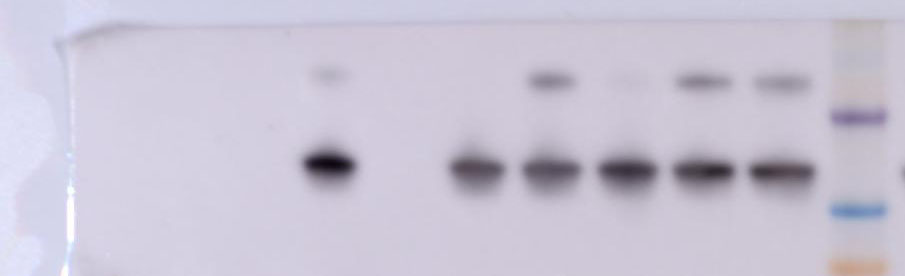

Supplement: Figure 1—source data 1. [file elife-89185-fig1-data1.zip › Figure 1-source data 1/Fig. 1E/2021.05.05_07.59.06-12_Ch+Marker_ATG3.jpg]

B

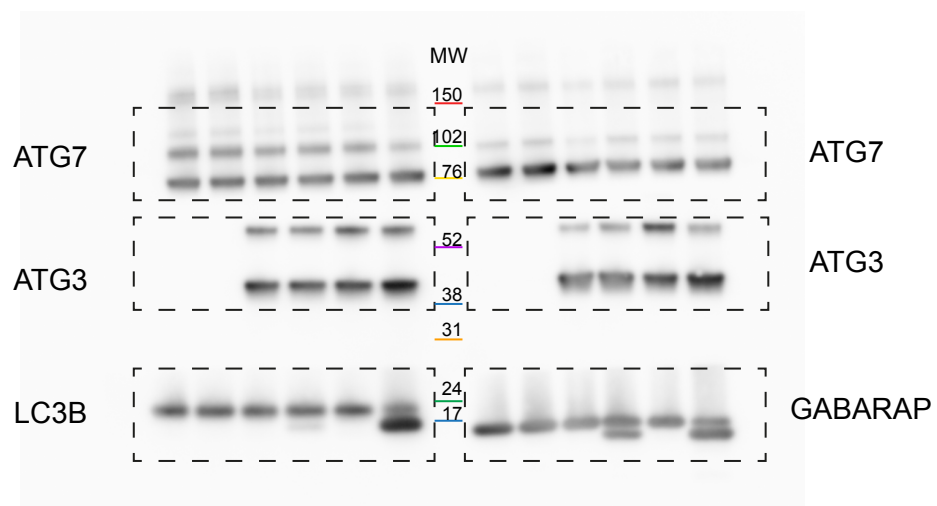

Figure 1-figure supplement 1

Supplement: Figure 1—figure supplement 1—source data 1. [file elife-89185-fig1-figsupp1-data1.zip › Figure 1-figure supplement 1-source data 1/Figure 1-S1-source data_lablled.pdf]

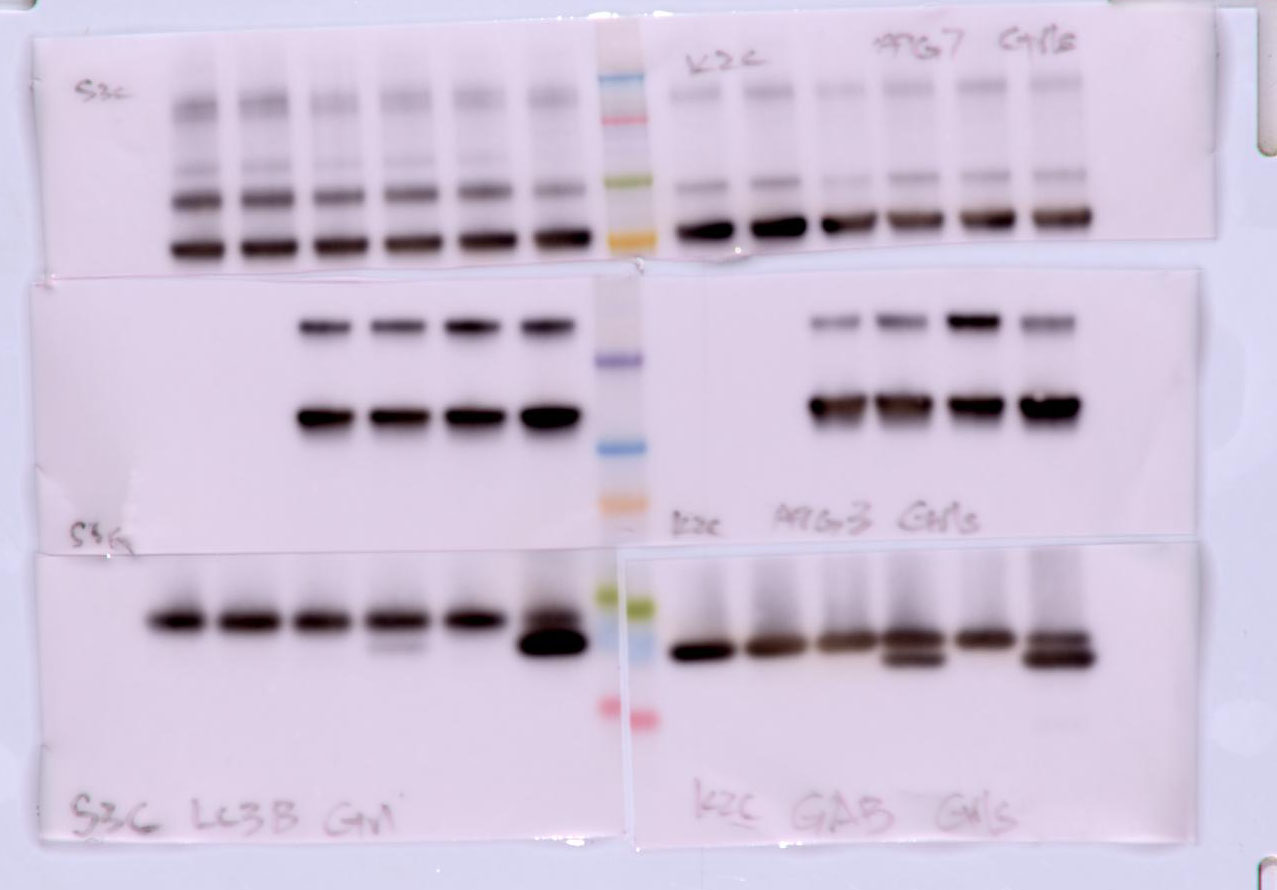

Supplement: Figure 1—figure supplement 1—source data 1. [file elife-89185-fig1-figsupp1-data1.zip › Figure 1-figure supplement 1-source data 1/Fig. 1-figure supplement 1B/2021.08.28_10.59.45_Ch+Marker.jpg]

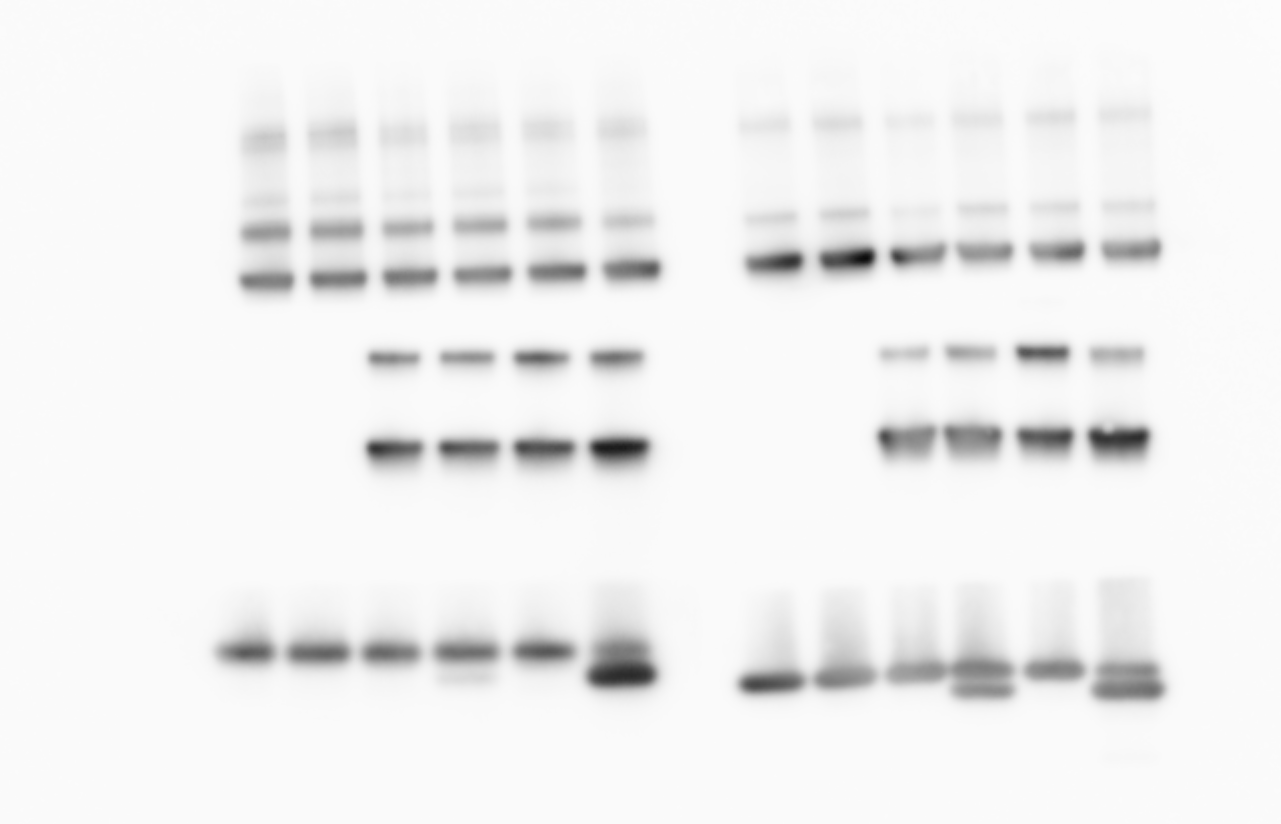

Supplement: Figure 1—figure supplement 1—source data 1. [file elife-89185-fig1-figsupp1-data1.zip › Figure 1-figure supplement 1-source data 1/Fig. 1-figure supplement 1B/2021.08.28_10.59.45_Ch.tif]

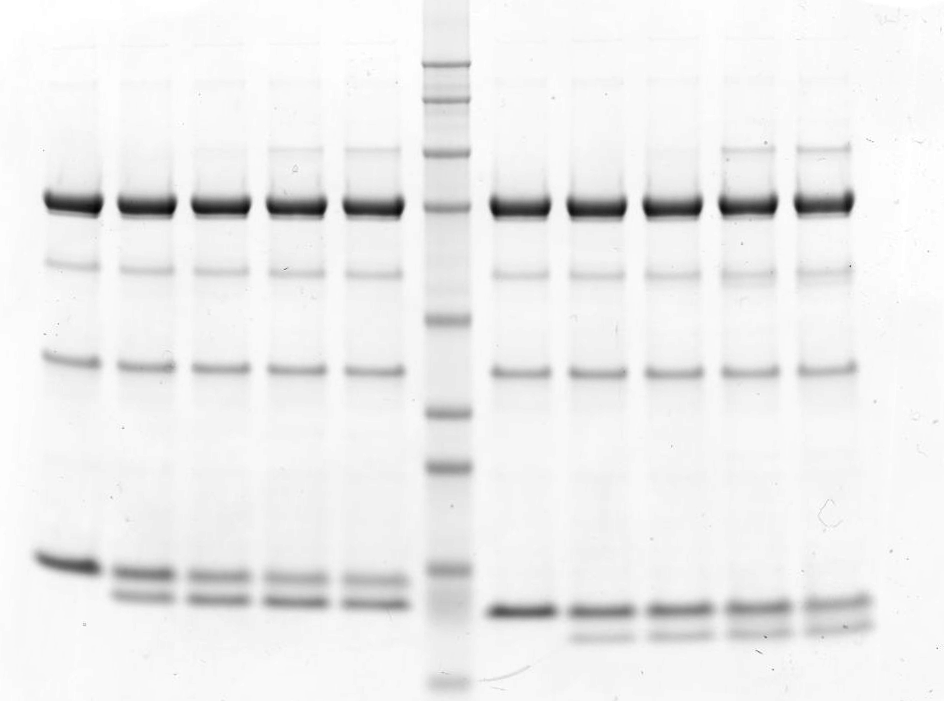

Supplement: Figure 1—figure supplement 2—source data 1. [file elife-89185-fig1-figsupp2-data1.zip › Figure 1-figure supplement 2-source data 1/Figure 1-S2B_LC3B WT delN ERlike lipidation.tif]

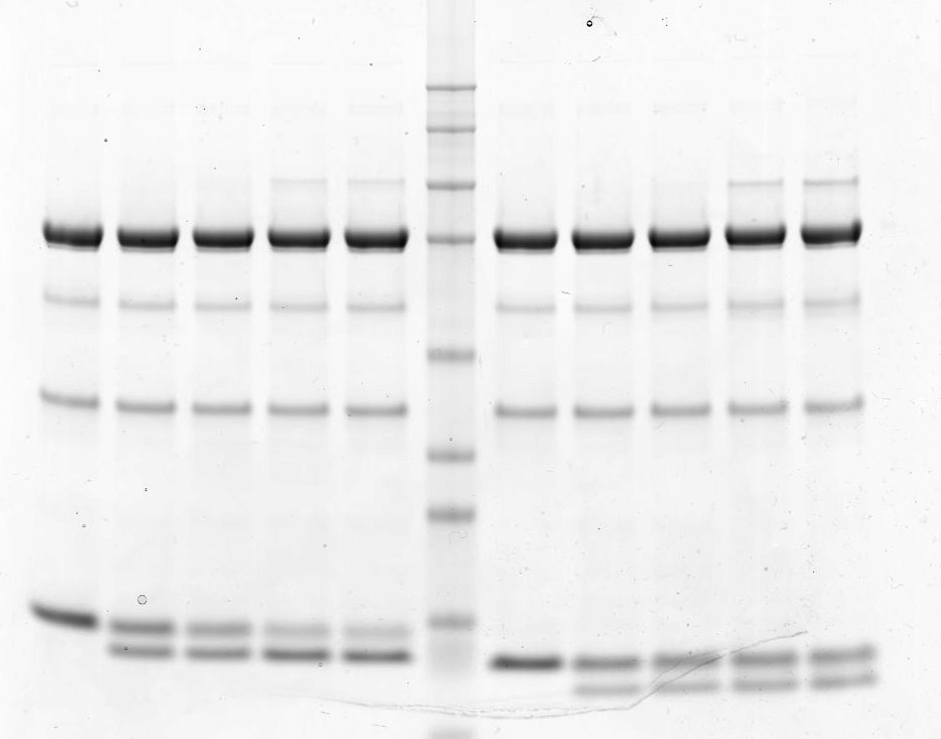

Supplement: Figure 1—figure supplement 2—source data 1. [file elife-89185-fig1-figsupp2-data1.zip › Figure 1-figure supplement 2-source data 1/Figure 1-S2A_LC3B WT delN11 50DOPE lipidation.tif]

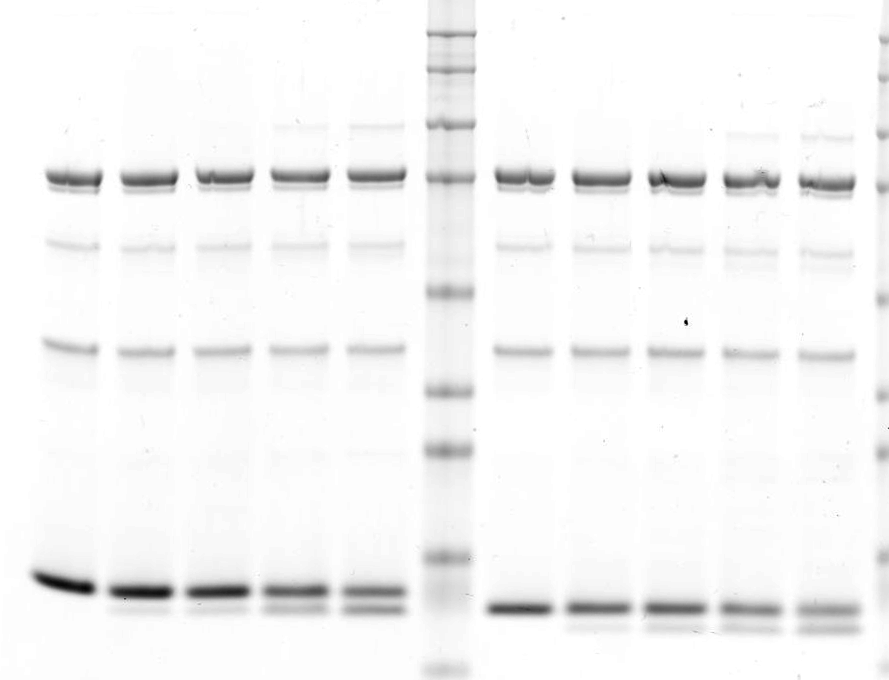

Supplement: Figure 1—figure supplement 2—source data 1. [file elife-89185-fig1-figsupp2-data1.zip › Figure 1-figure supplement 2-source data 1/Figure 1-S2D_GAB WT delN9 ERlike lipidation.tif]

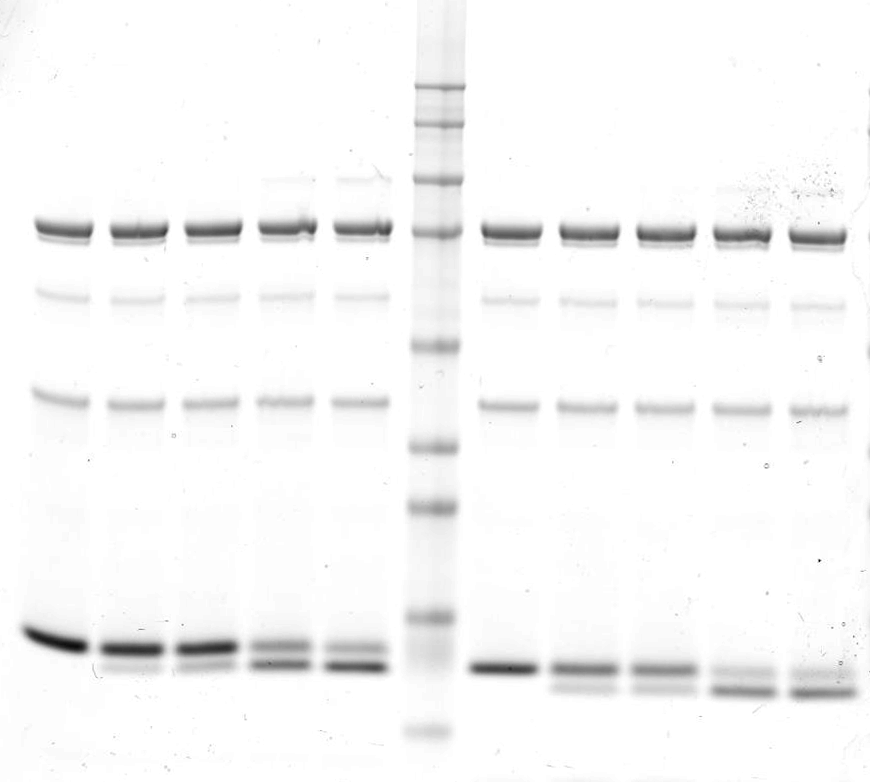

Supplement: Figure 1—figure supplement 2—source data 1. [file elife-89185-fig1-figsupp2-data1.zip › Figure 1-figure supplement 2-source data 1/Figure 1-S2C_GAB WT delN9 50DOPE lipidation.tif]

B

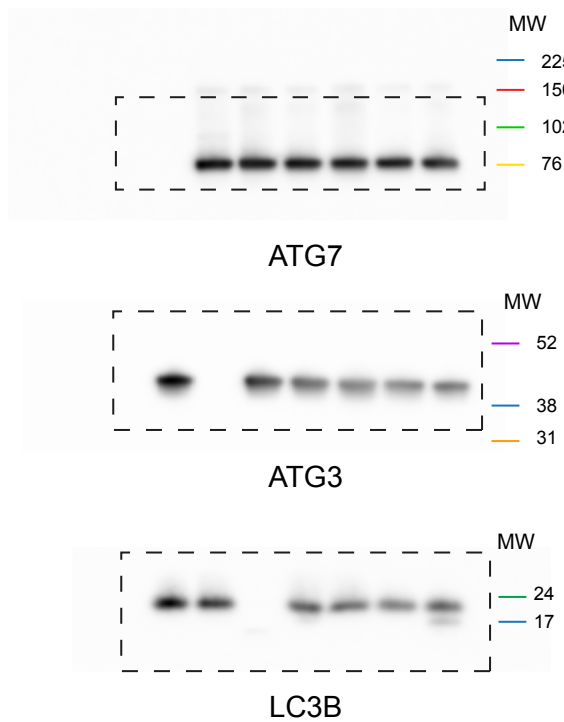

C

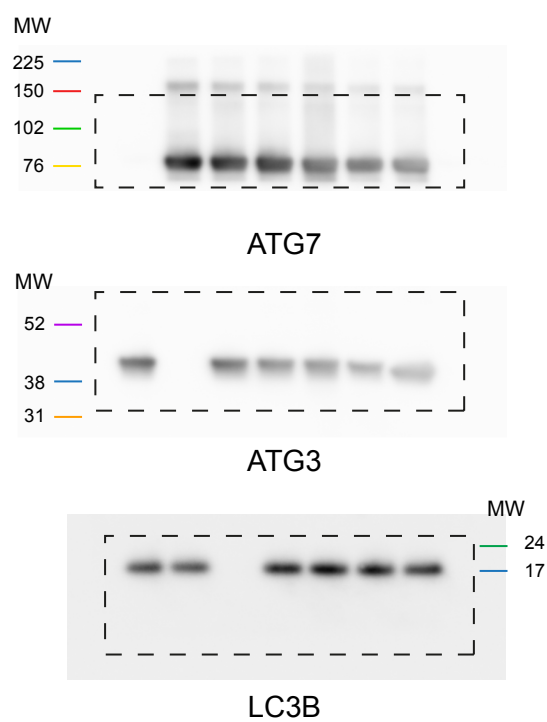

D

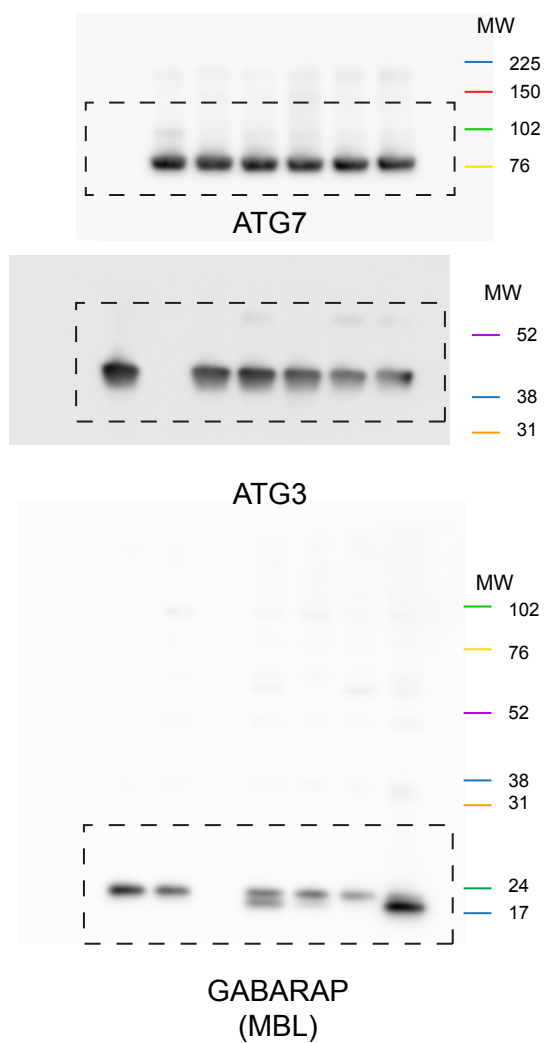

E

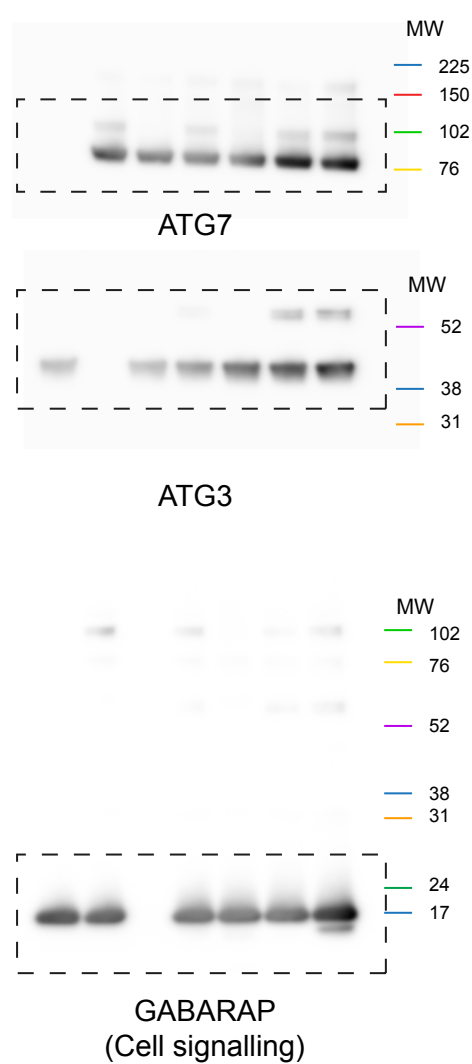

Figure 5

Supplement: Figure 5—source data 1. [file elife-89185-fig5-data1.zip › Figure 5-source data 1/Figure 5-source data _labelled.pdf]

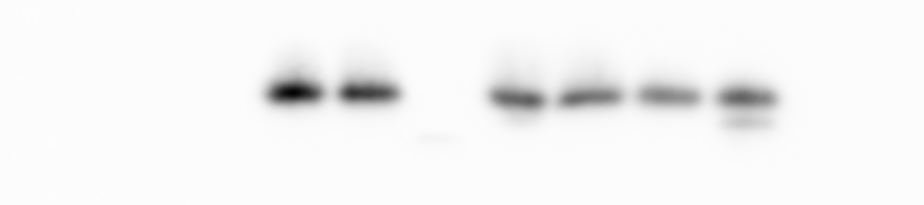

Supplement: Figure 5—source data 1. [file elife-89185-fig5-data1.zip › Figure 5-source data 1/Fig. 5B/2022.03.15_13.01.31-10_Ch_LC3B.tif]

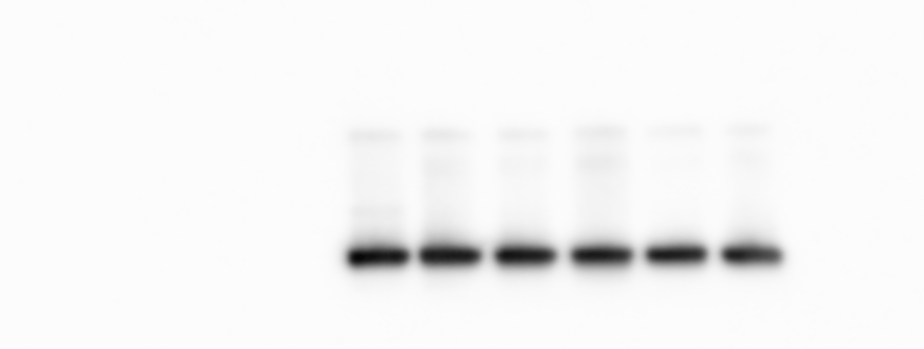

Supplement: Figure 5—source data 1. [file elife-89185-fig5-data1.zip › Figure 5-source data 1/Fig. 5B/2022.03.15_13.01.31-10_Ch_ATG7.tif]

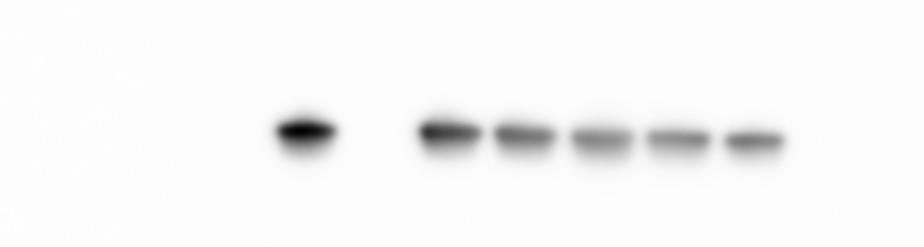

Supplement: Figure 5—source data 1. [file elife-89185-fig5-data1.zip › Figure 5-source data 1/Fig. 5B/2022.03.15_13.01.31-10_Ch_ATG3.tif]

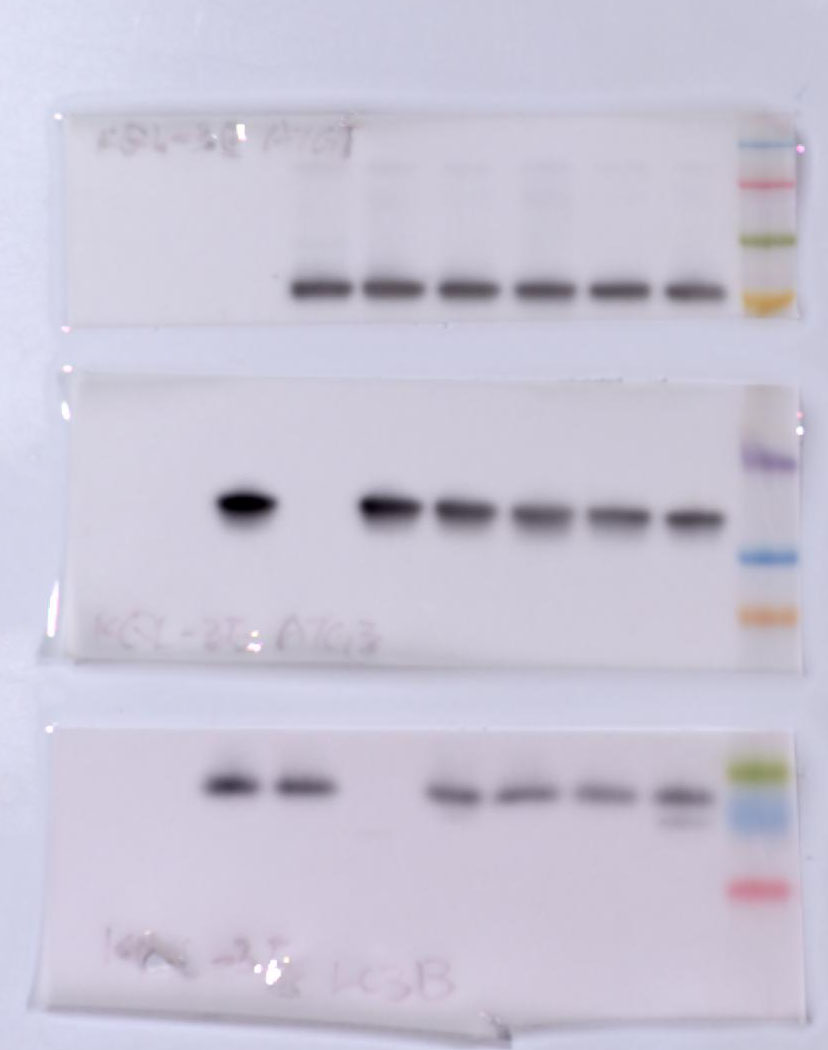

Supplement: Figure 5—source data 1. [file elife-89185-fig5-data1.zip › Figure 5-source data 1/Fig. 5B/2022.03.15_13.01.31-10_Ch+Marker.jpg]

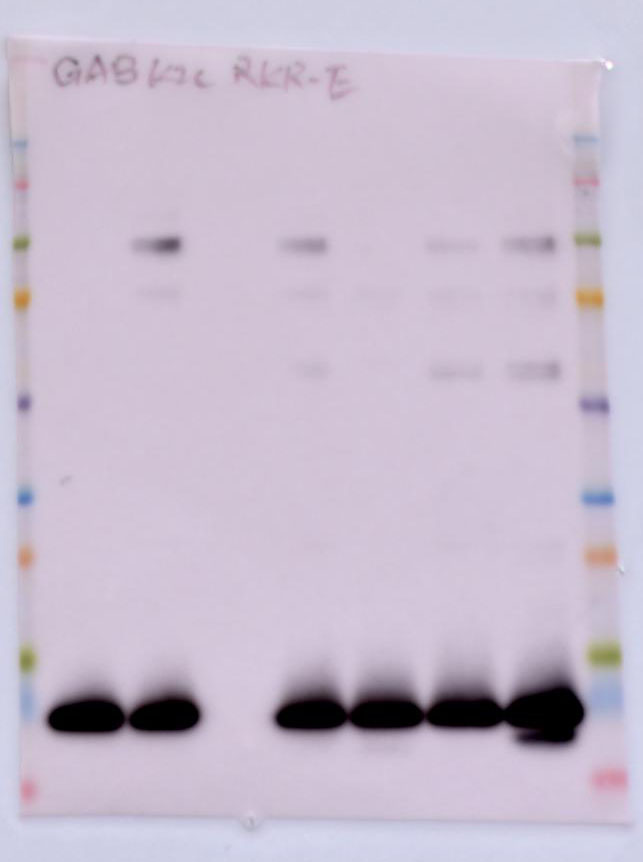

Supplement: Figure 5—source data 1. [file elife-89185-fig5-data1.zip › Figure 5-source data 1/Fig. 5E/2021.06.30_07.21.01_Ch+Marker_GABARAP_CellSig.jpg]

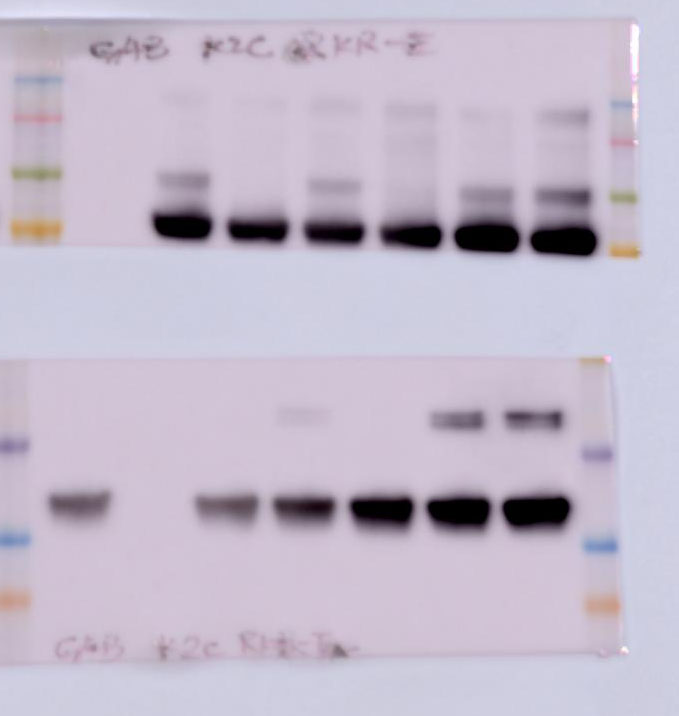

Supplement: Figure 5—source data 1. [file elife-89185-fig5-data1.zip › Figure 5-source data 1/Fig. 5E/2021.06.30_07.09.59_Ch+Marker_ATG7_ATG3.jpg]

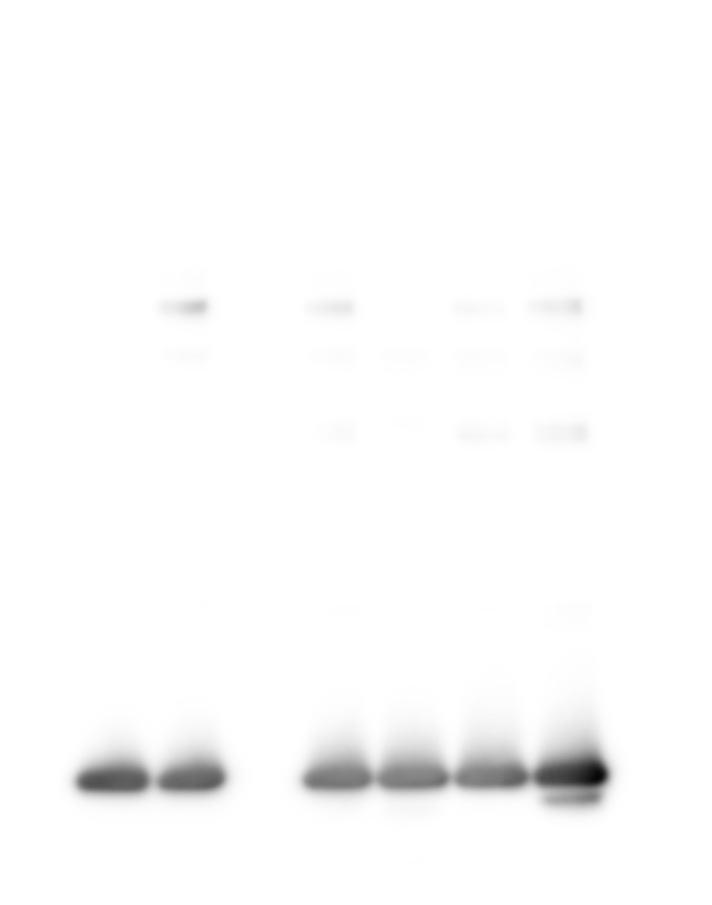

Supplement: Figure 5—source data 1. [file elife-89185-fig5-data1.zip › Figure 5-source data 1/Fig. 5E/2021.06.30_07.21.01_Ch_GABARAP_CellSig.tif]

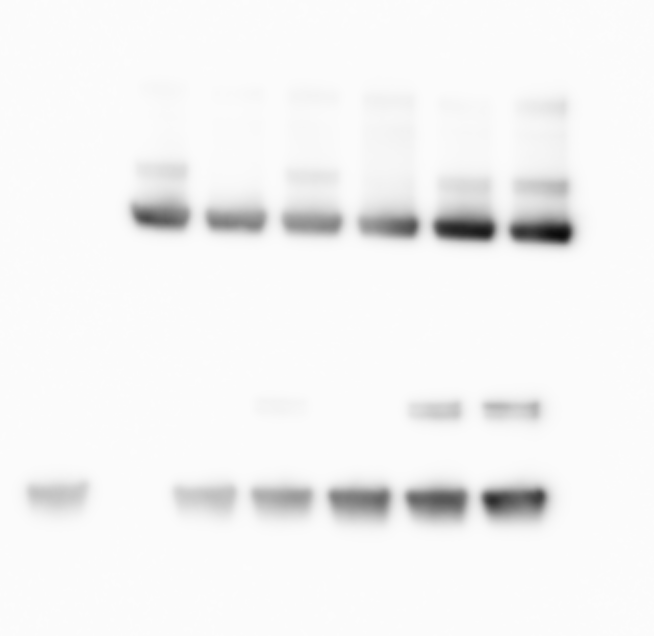

Supplement: Figure 5—source data 1. [file elife-89185-fig5-data1.zip › Figure 5-source data 1/Fig. 5E/2021.06.30_07.09.59_Ch_ATG7_ATG3.tif]

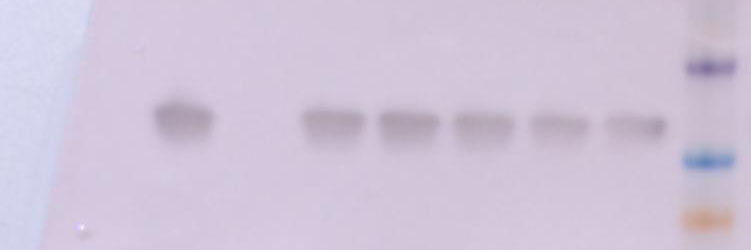

Supplement: Figure 5—source data 1. [file elife-89185-fig5-data1.zip › Figure 5-source data 1/Fig. 5D/2021.07.22_13.55.55_Ch+Marker_ATG3.jpg]

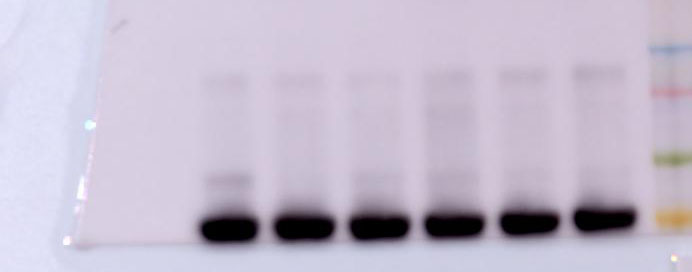

Supplement: Figure 5—source data 1. [file elife-89185-fig5-data1.zip › Figure 5-source data 1/Fig. 5D/2021.07.22_13.55.55_Ch+Marker_ATG7.jpg]

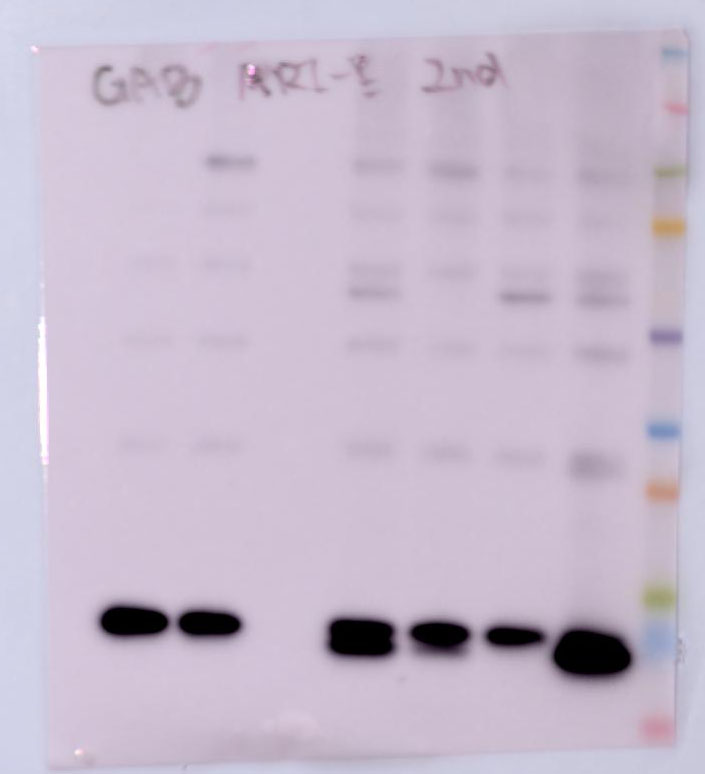

Supplement: Figure 5—source data 1. [file elife-89185-fig5-data1.zip › Figure 5-source data 1/Fig. 5D/2021.12.01_08.55.10_Ch+Marker_GAB_MBL.jpg]

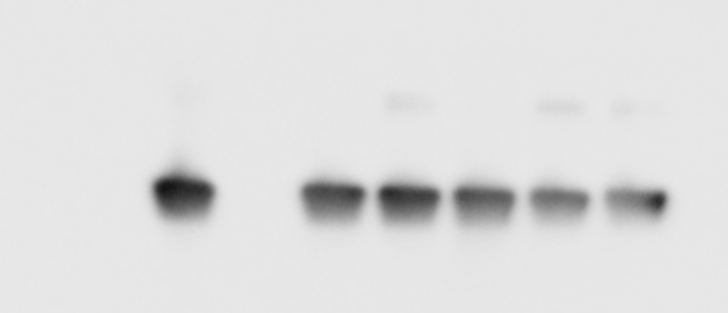

Supplement: Figure 5—source data 1. [file elife-89185-fig5-data1.zip › Figure 5-source data 1/Fig. 5D/2021.07.22_13.55.55_Ch_ATG3.tif]

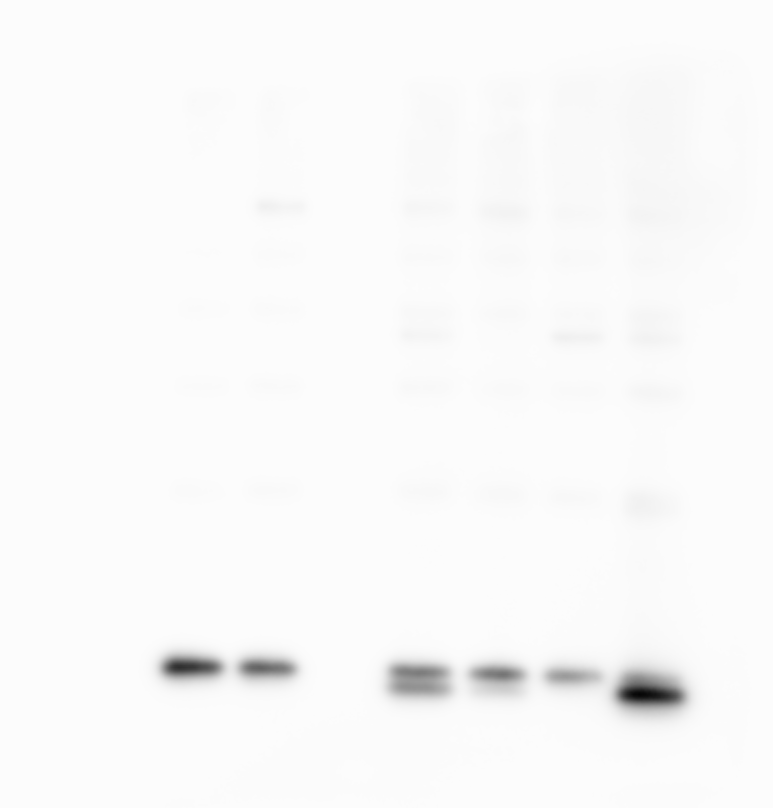

Supplement: Figure 5—source data 1. [file elife-89185-fig5-data1.zip › Figure 5-source data 1/Fig. 5D/2021.12.01_08.55.10_Ch_GAB_MBL.tif]

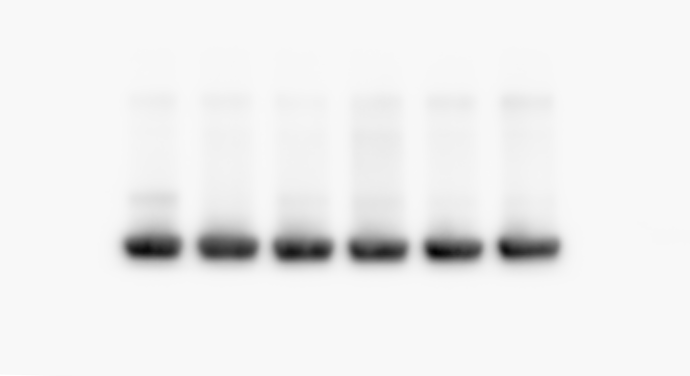

Supplement: Figure 5—source data 1. [file elife-89185-fig5-data1.zip › Figure 5-source data 1/Fig. 5D/2021.07.22_13.55.55_Ch_ATG7.tif]

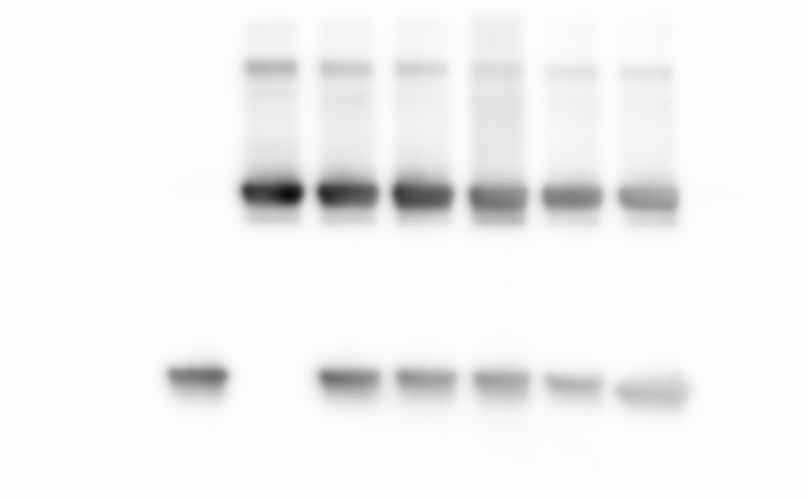

Supplement: Figure 5—source data 1. [file elife-89185-fig5-data1.zip › Figure 5-source data 1/Fig. 5C/2021.06.09_09.32.37_Ch_ATG7_ATG3.tif]

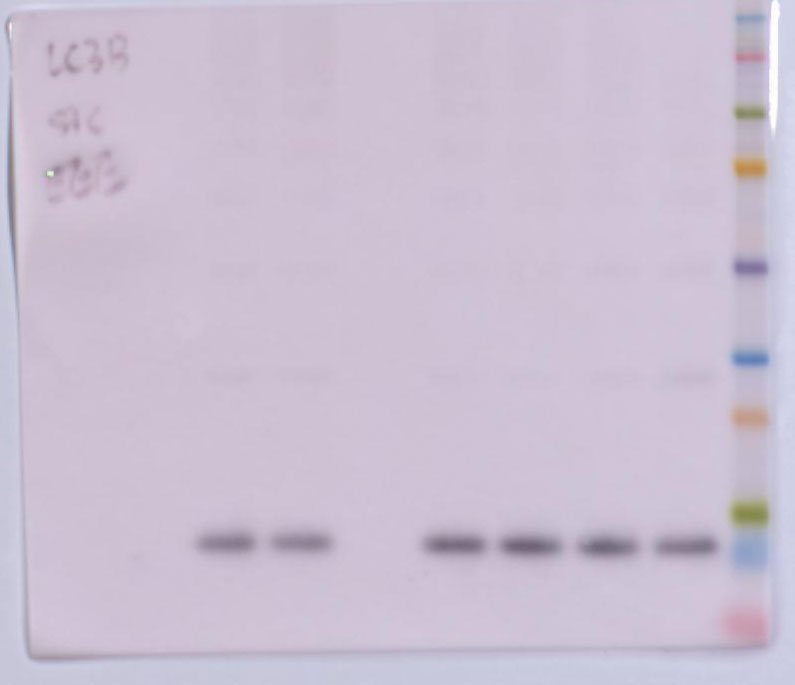

Supplement: Figure 5—source data 1. [file elife-89185-fig5-data1.zip › Figure 5-source data 1/Fig. 5C/2021.06.09_09.32.37_Ch+Marker_LC3B.jpg]

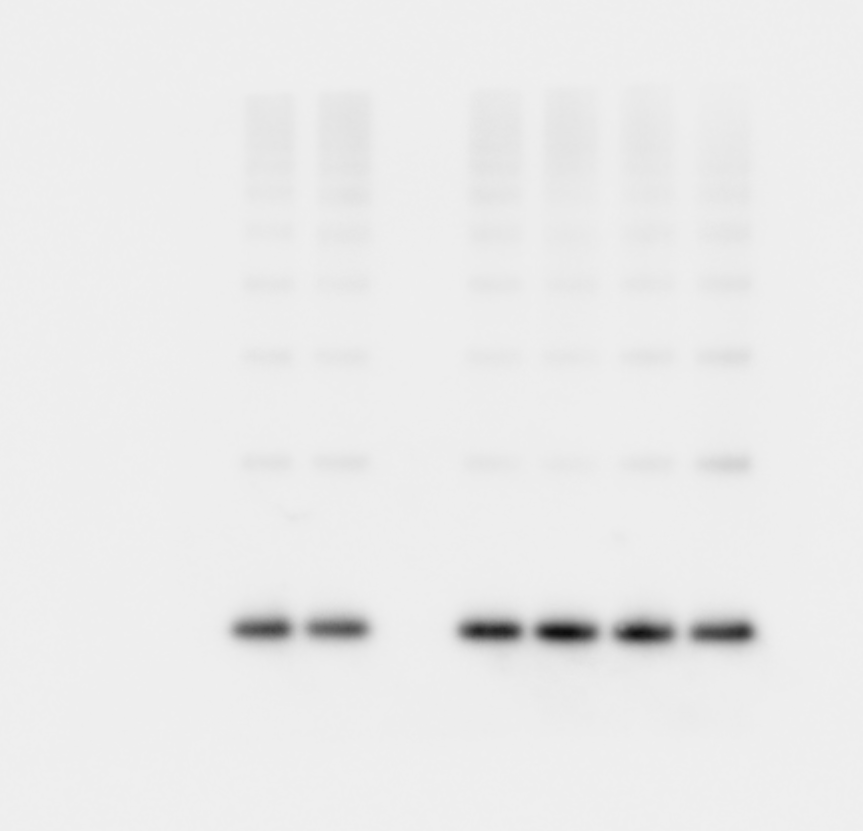

Supplement: Figure 5—source data 1. [file elife-89185-fig5-data1.zip › Figure 5-source data 1/Fig. 5C/2021.06.09_09.32.37_Ch_LC3B.tif]

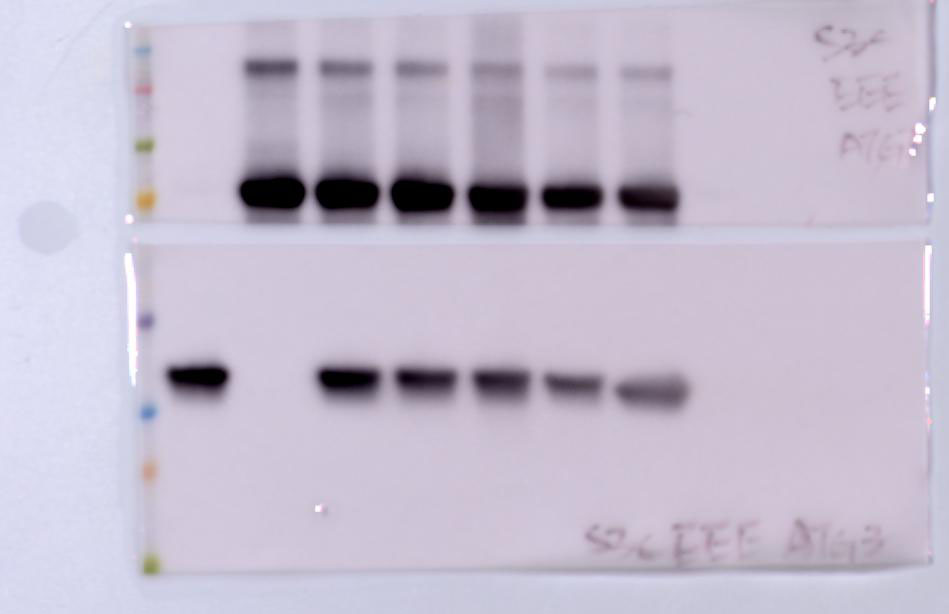

Supplement: Figure 5—source data 1. [file elife-89185-fig5-data1.zip › Figure 5-source data 1/Fig. 5C/2021.06.09_09.32.37_Ch+Marker_ATG7_ATG3.jpg]

A

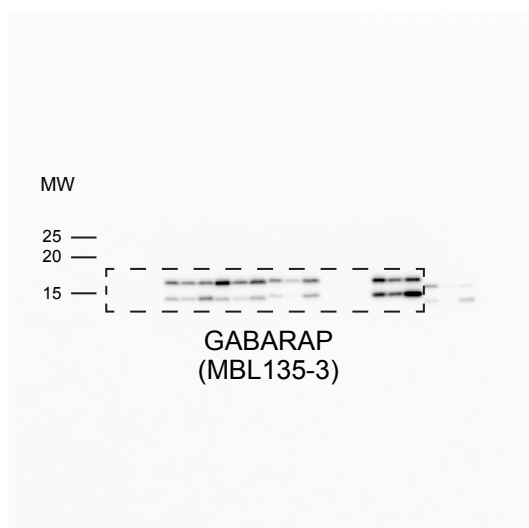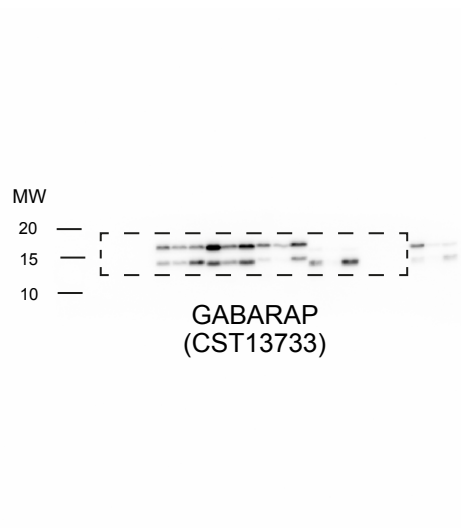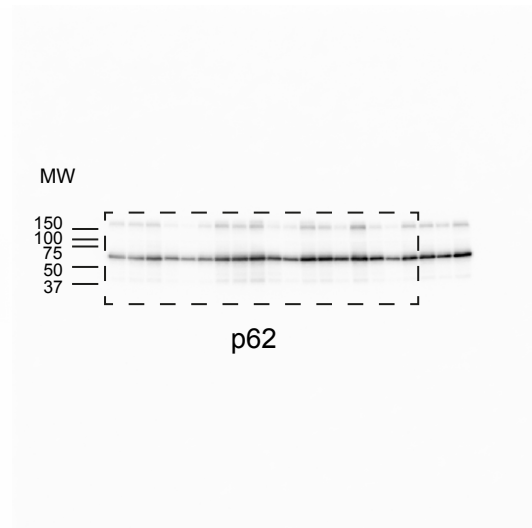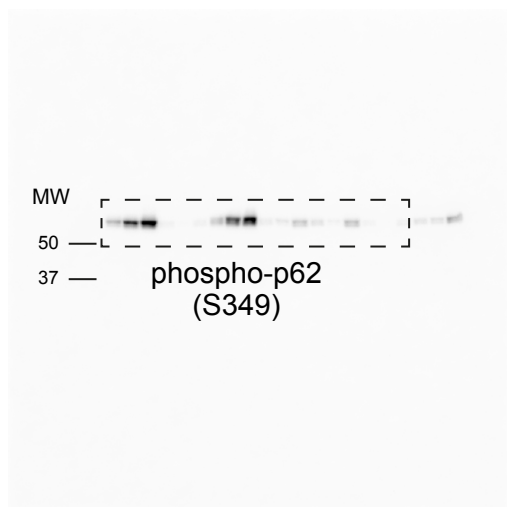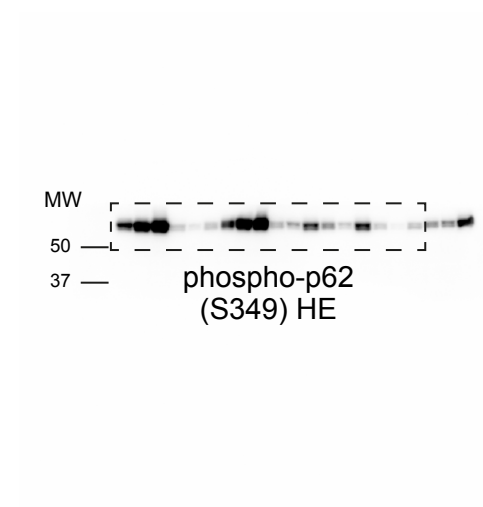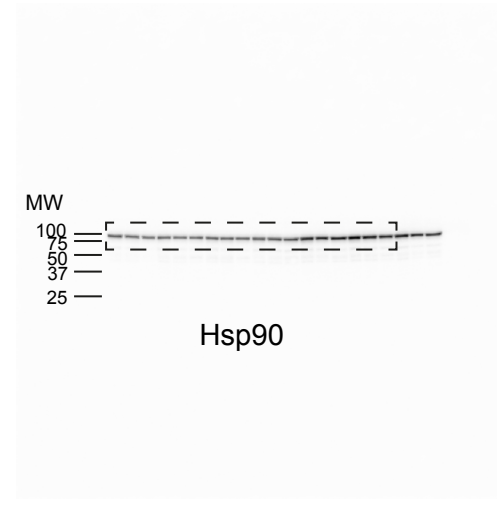

F

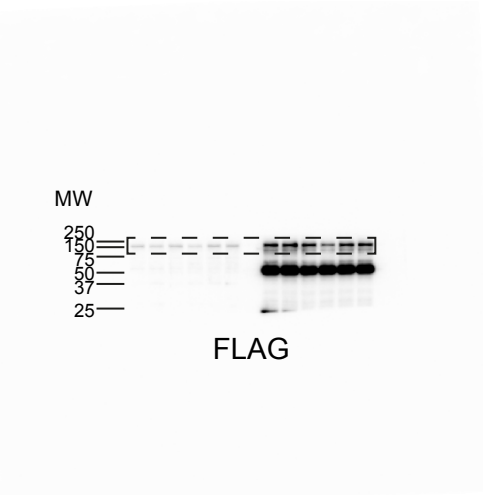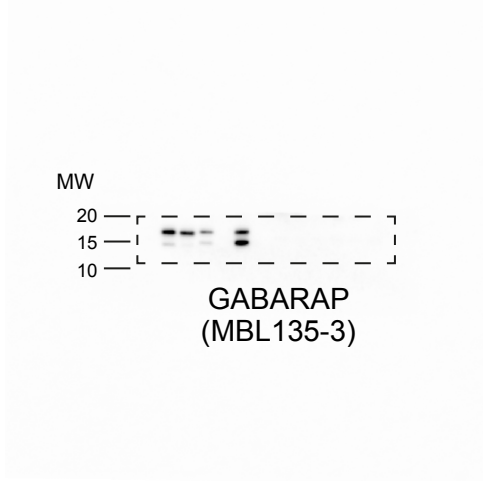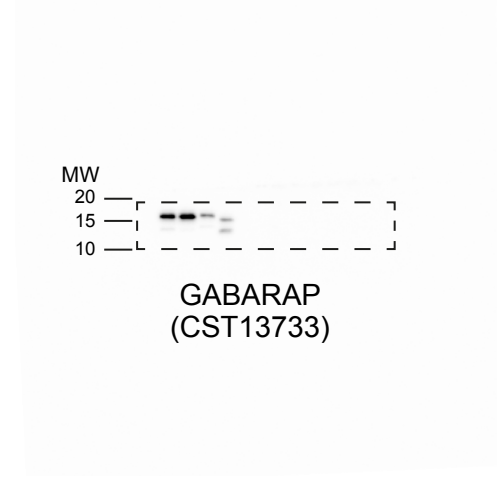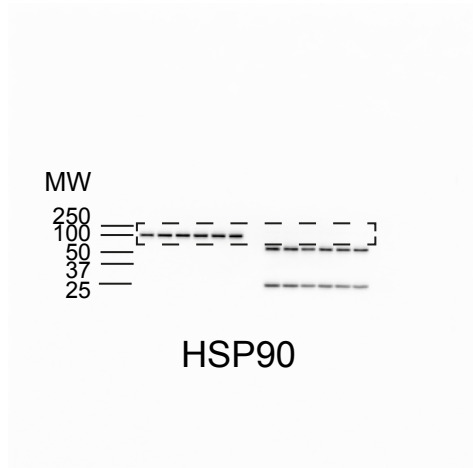

Figure 6

Supplement: Figure 6—source data 1. [file elife-89185-fig6-data1.zip › Figure 6-source data 1/Figure 6-source data_labelled.pdf]

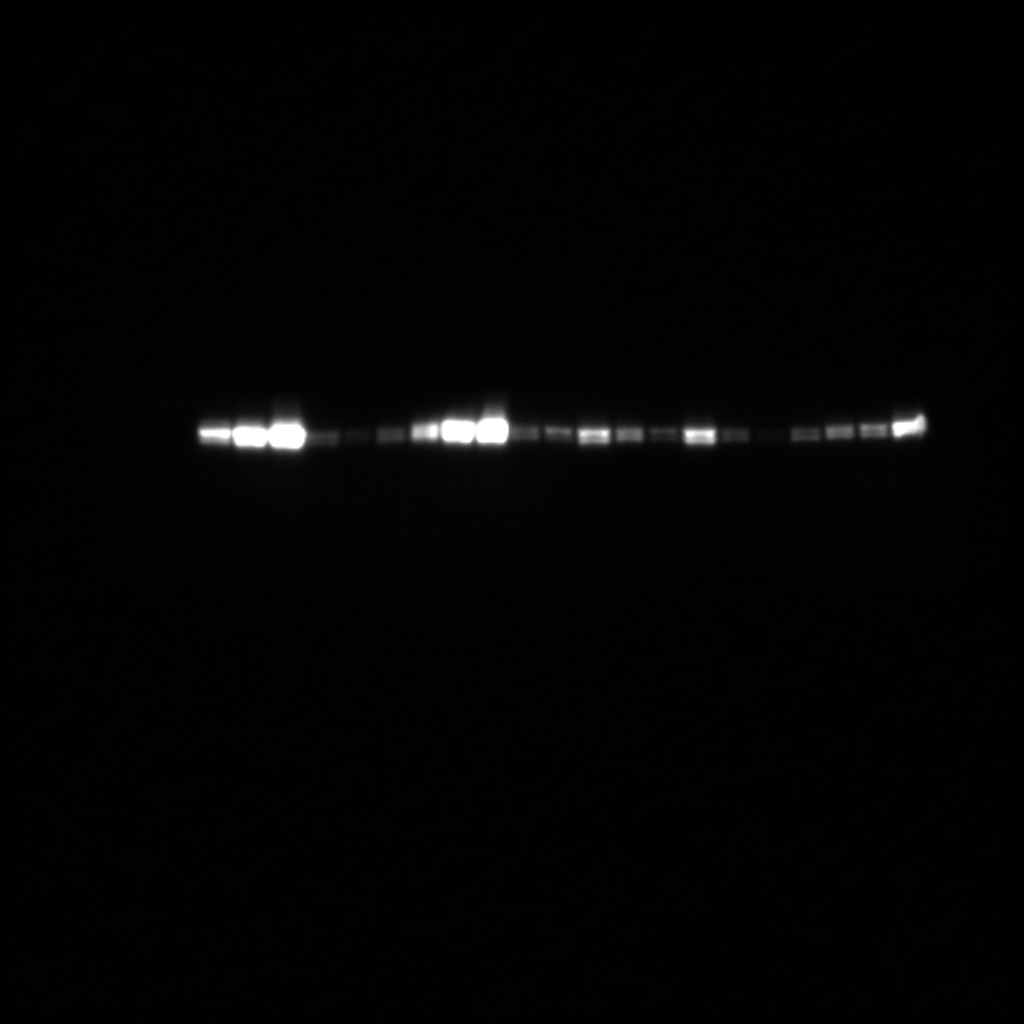

Supplement: Figure 6—source data 1. [file elife-89185-fig6-data1.zip › Figure 6-source data 1/Fig. 6A/IM010752_2 (phospho-p62 HE).TIF]

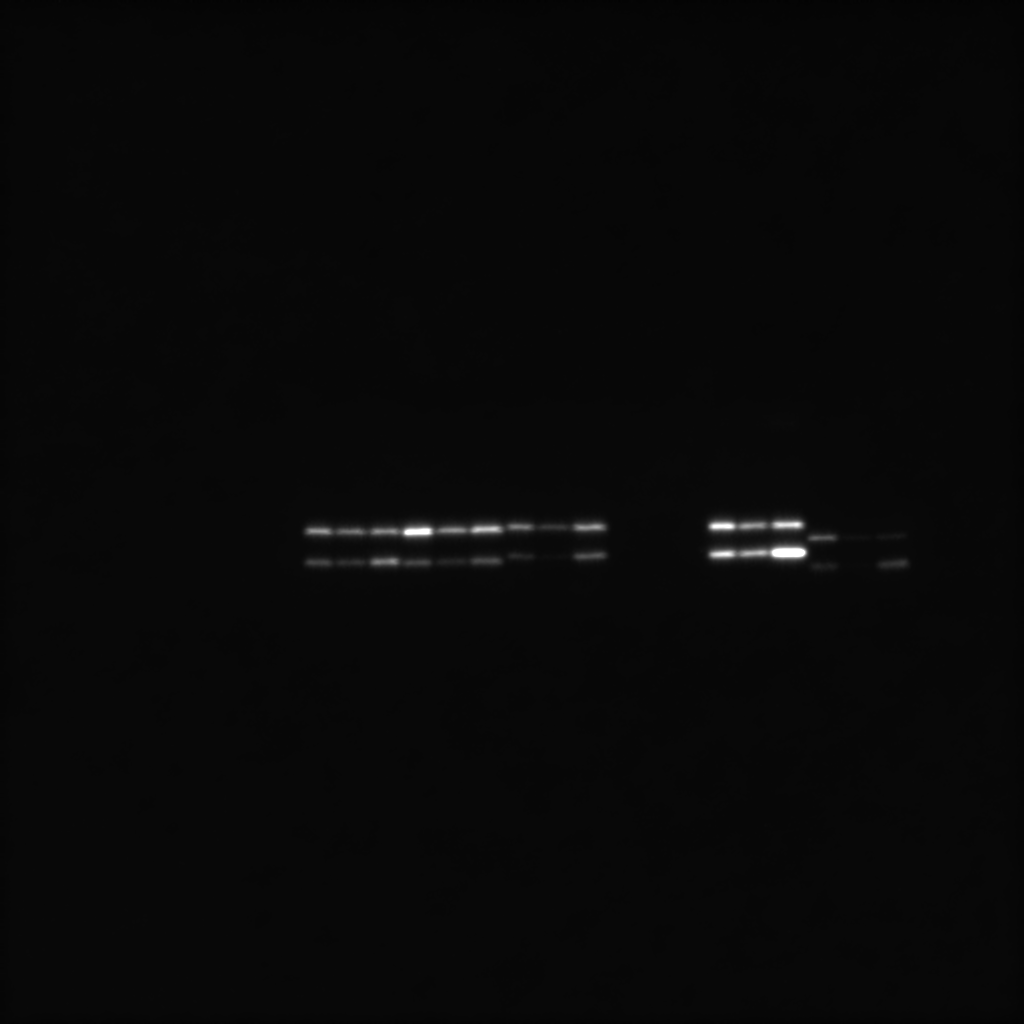

Supplement: Figure 6—source data 1. [file elife-89185-fig6-data1.zip › Figure 6-source data 1/Fig. 6A/IM010744_10Sum (GABARAP MBL135-3).TIF]

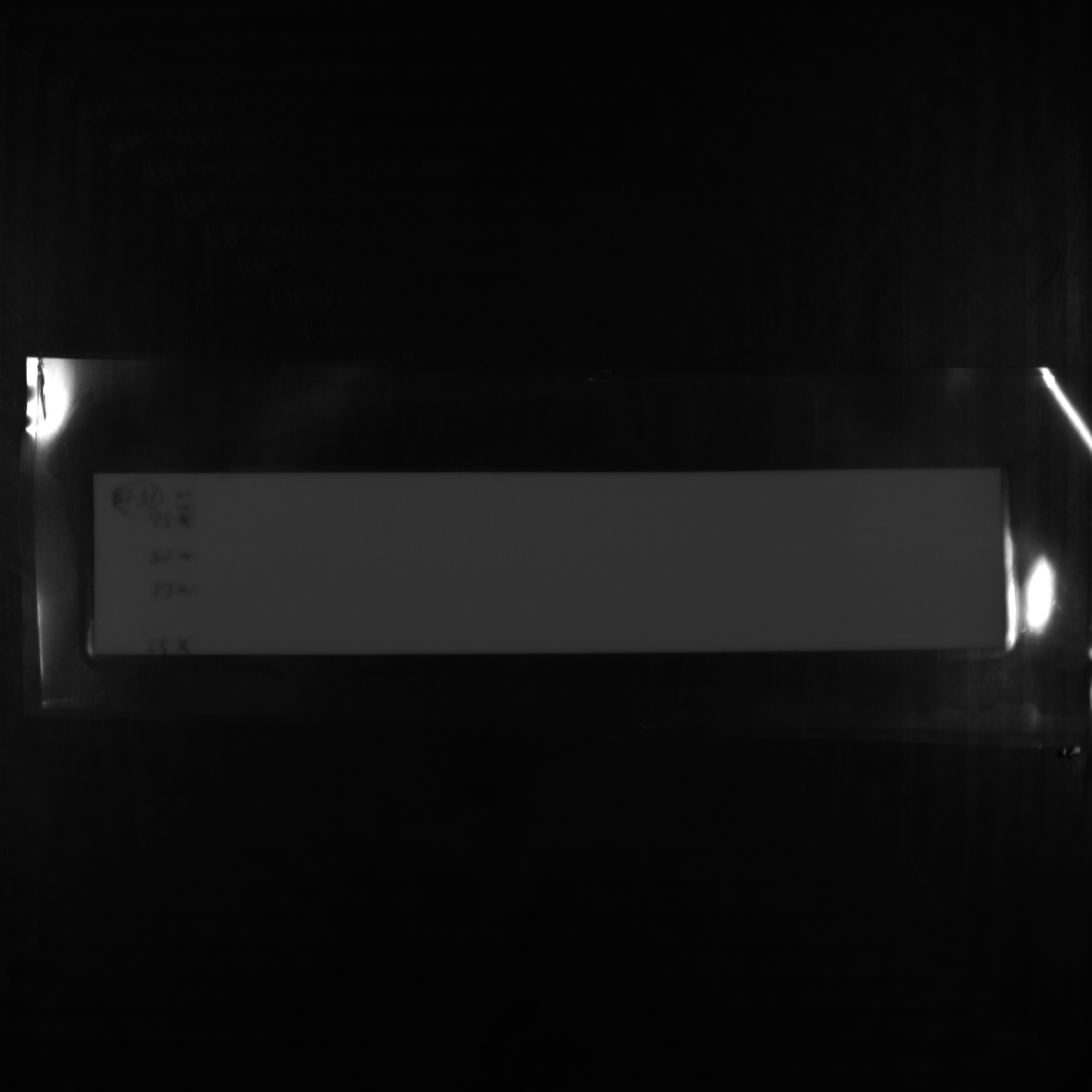

Supplement: Figure 6—source data 1. [file elife-89185-fig6-data1.zip › Figure 6-source data 1/Fig. 6A/IM010775_marker (HSP90).Tif]

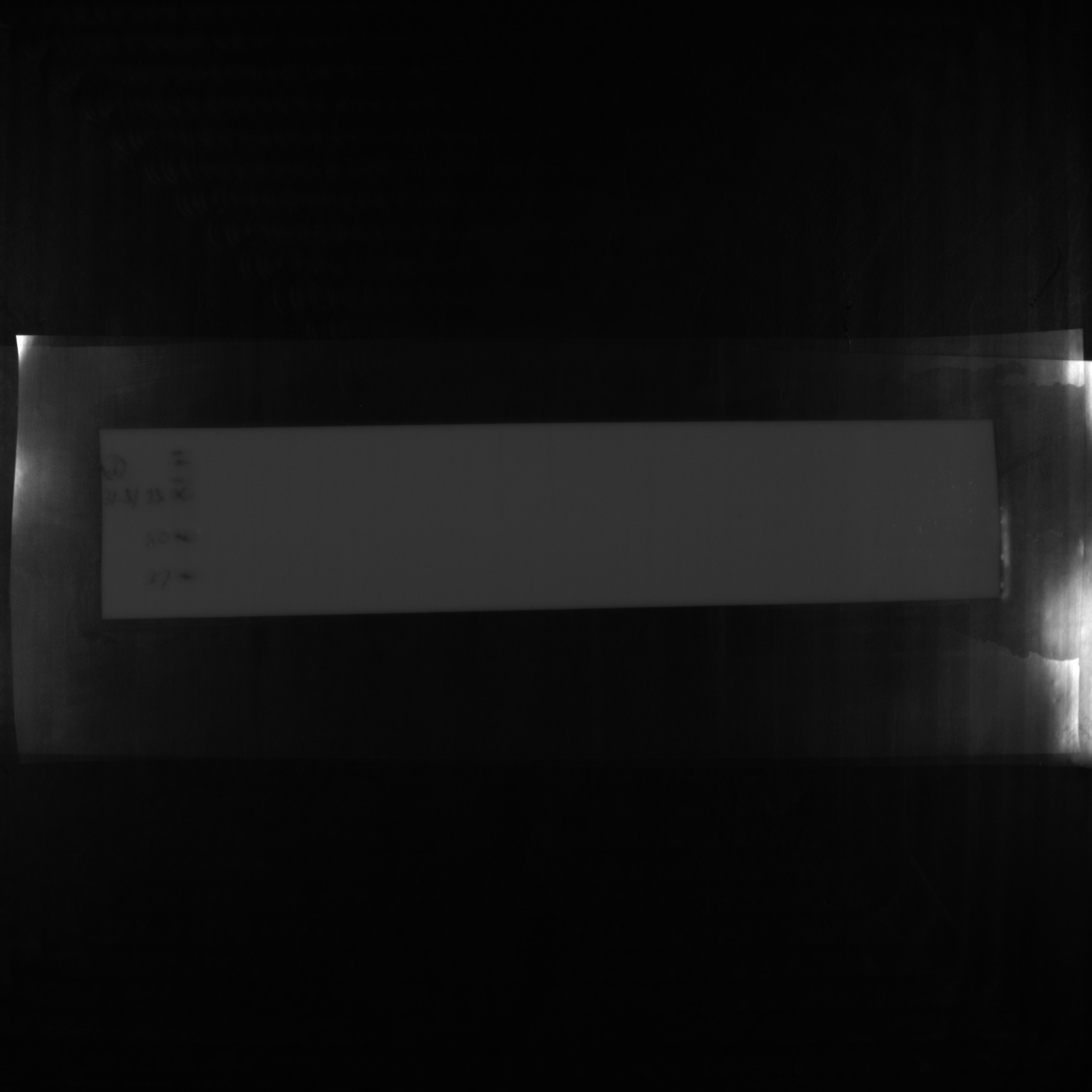

Supplement: Figure 6—source data 1. [file elife-89185-fig6-data1.zip › Figure 6-source data 1/Fig. 6A/IM010755_marker (p62).Tif]

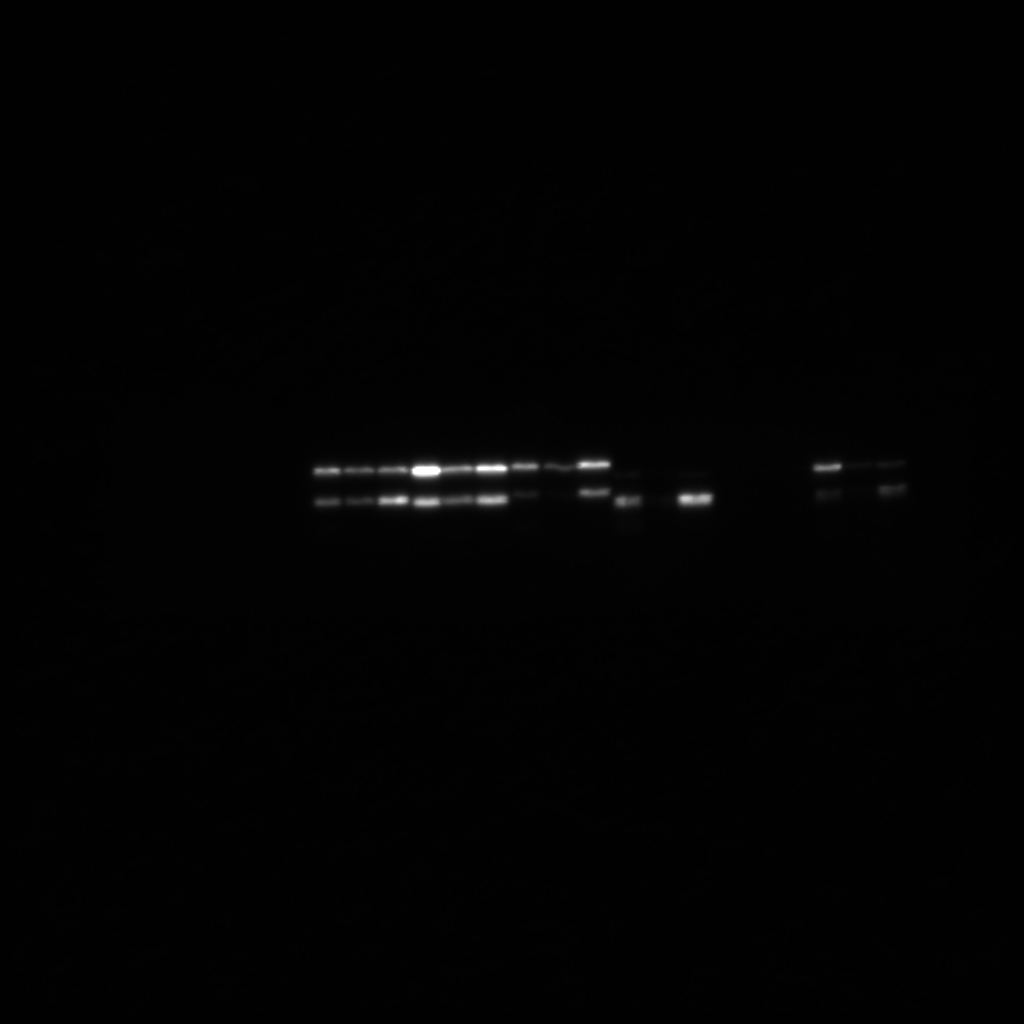

Supplement: Figure 6—source data 1. [file elife-89185-fig6-data1.zip › Figure 6-source data 1/Fig. 6A/IM010774_1 (GABARAP-CST13733).TIF]

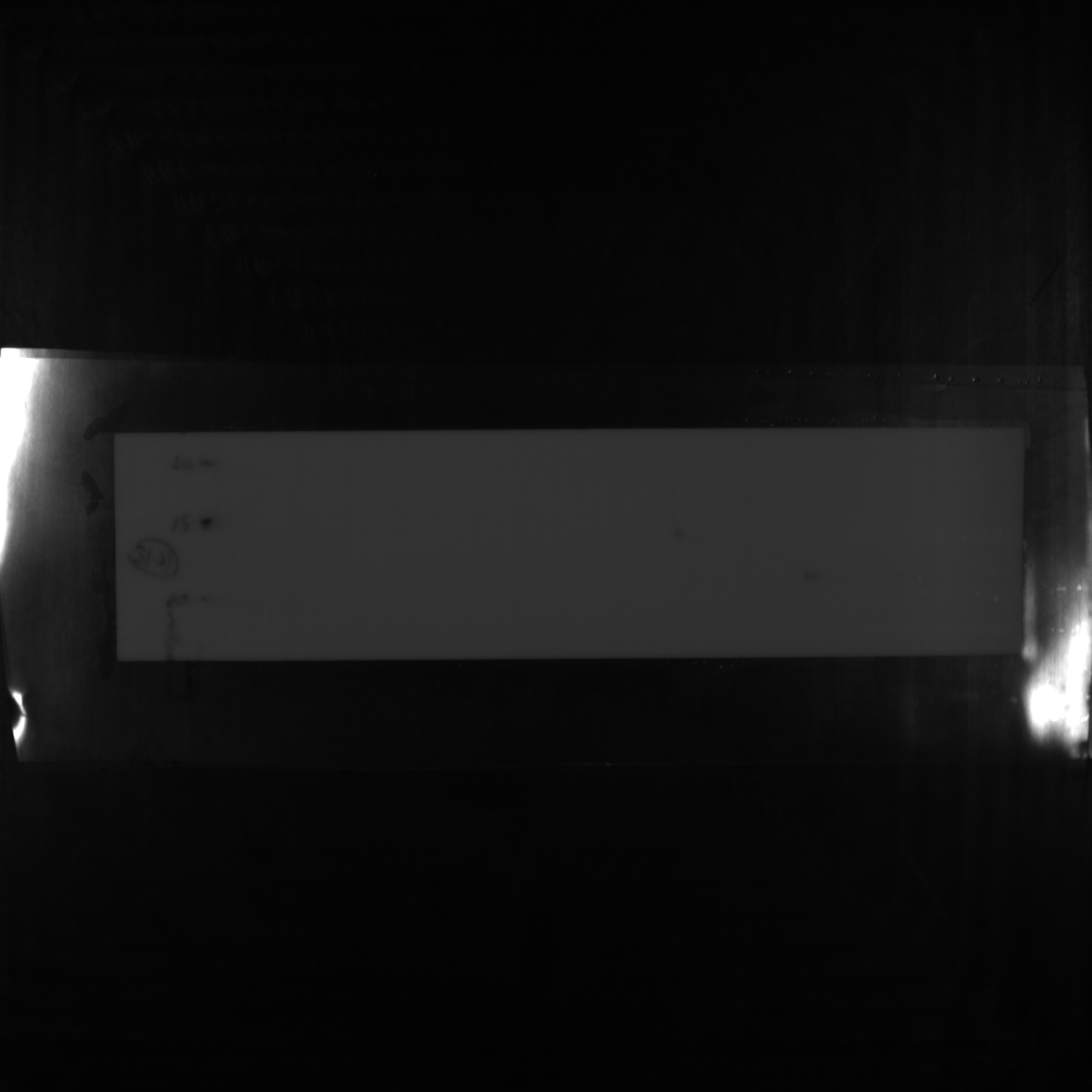

Supplement: Figure 6—source data 1. [file elife-89185-fig6-data1.zip › Figure 6-source data 1/Fig. 6A/IM010772_marker (GABARAP-CST13733).Tif]

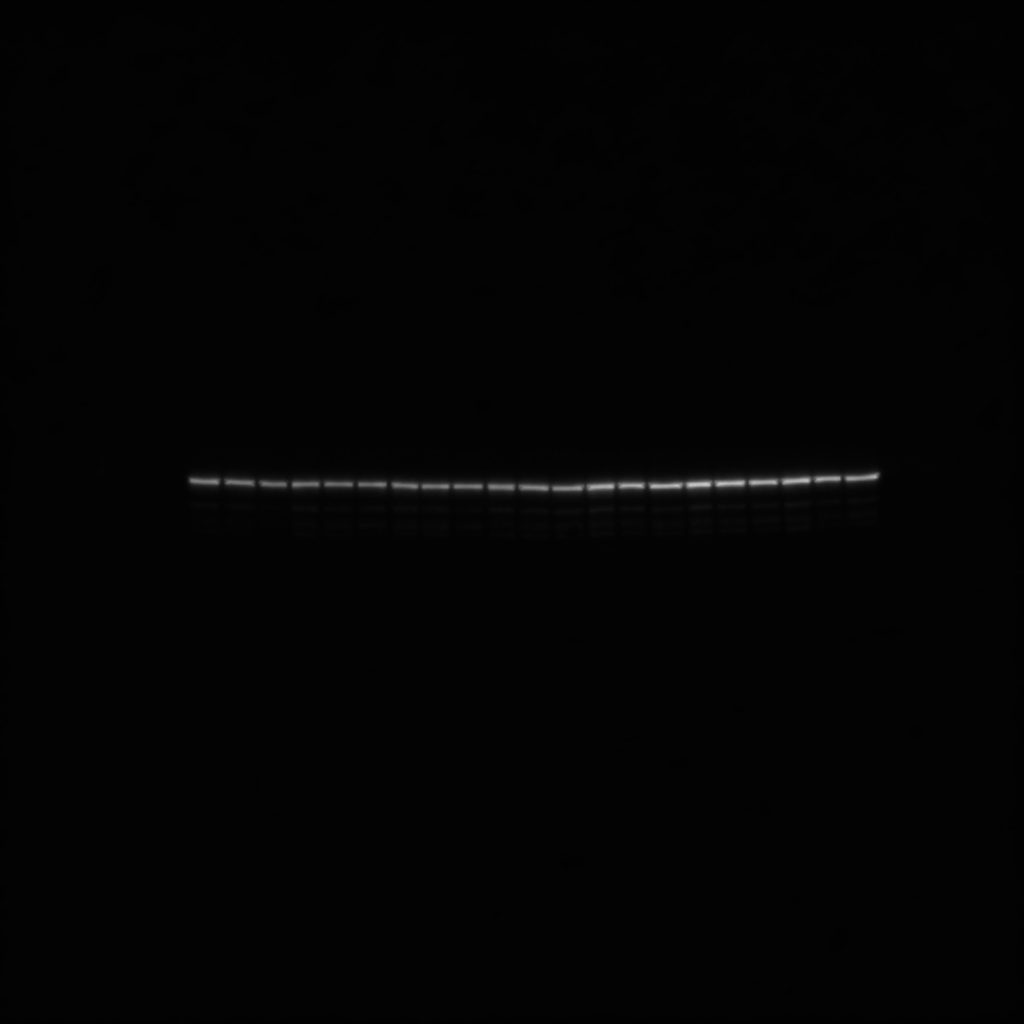

Supplement: Figure 6—source data 1. [file elife-89185-fig6-data1.zip › Figure 6-source data 1/Fig. 6A/IM010776_3Sum (HSP90).TIF]

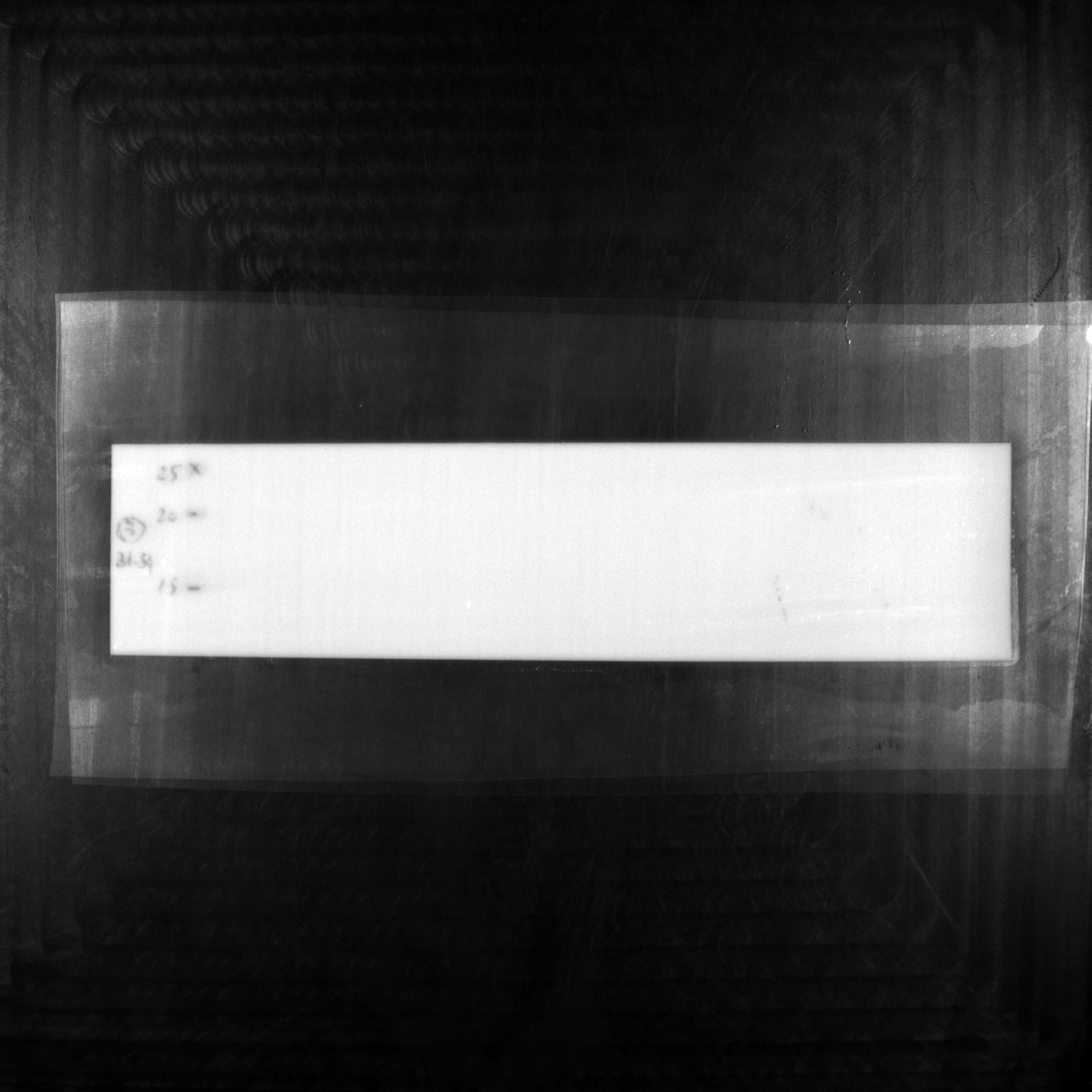

Supplement: Figure 6—source data 1. [file elife-89185-fig6-data1.zip › Figure 6-source data 1/Fig. 6A/IM010743_marker (GABARAP MBL135-3).Tif]

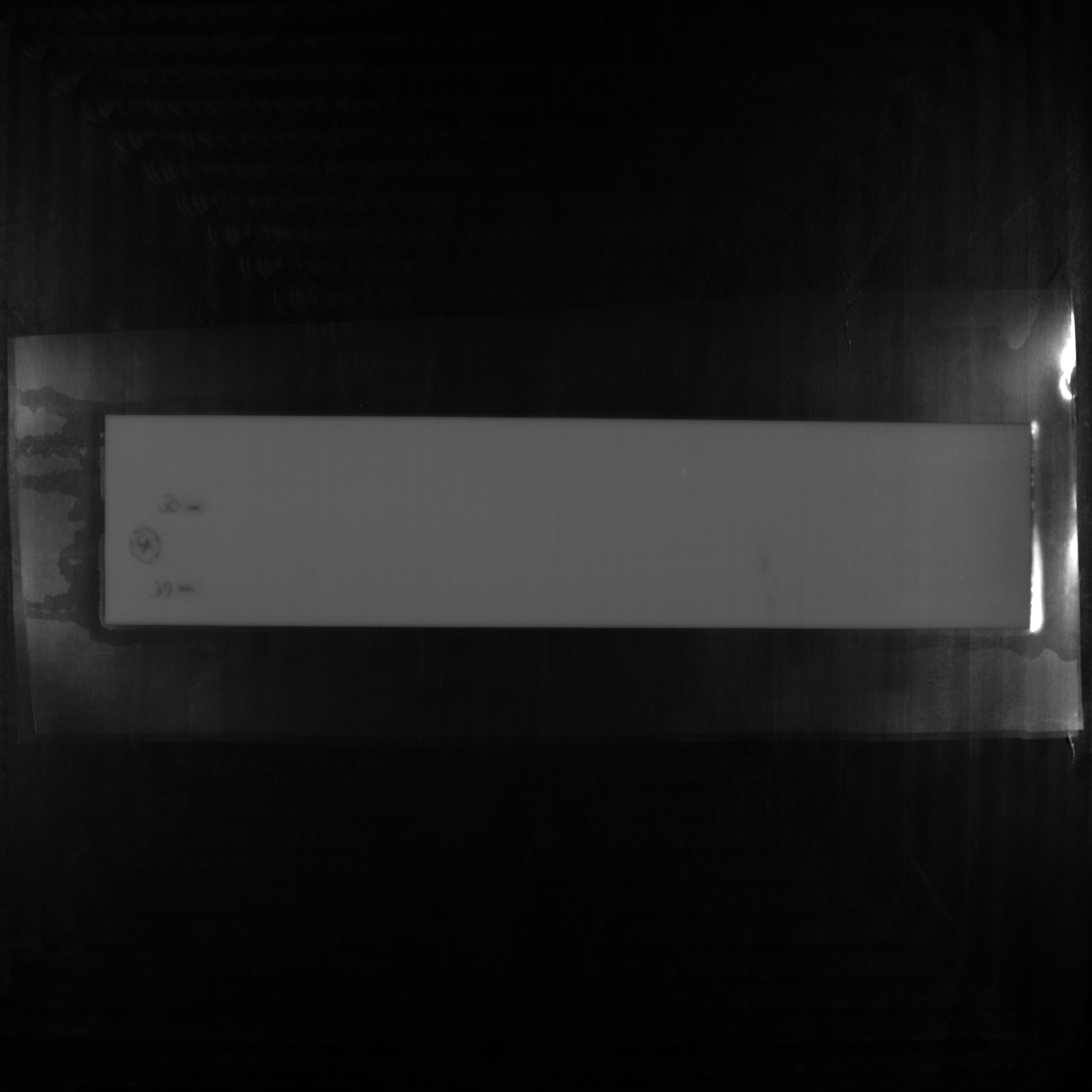

Supplement: Figure 6—source data 1. [file elife-89185-fig6-data1.zip › Figure 6-source data 1/Fig. 6A/IM010750_marker (phospho-p62 HE).Tif]

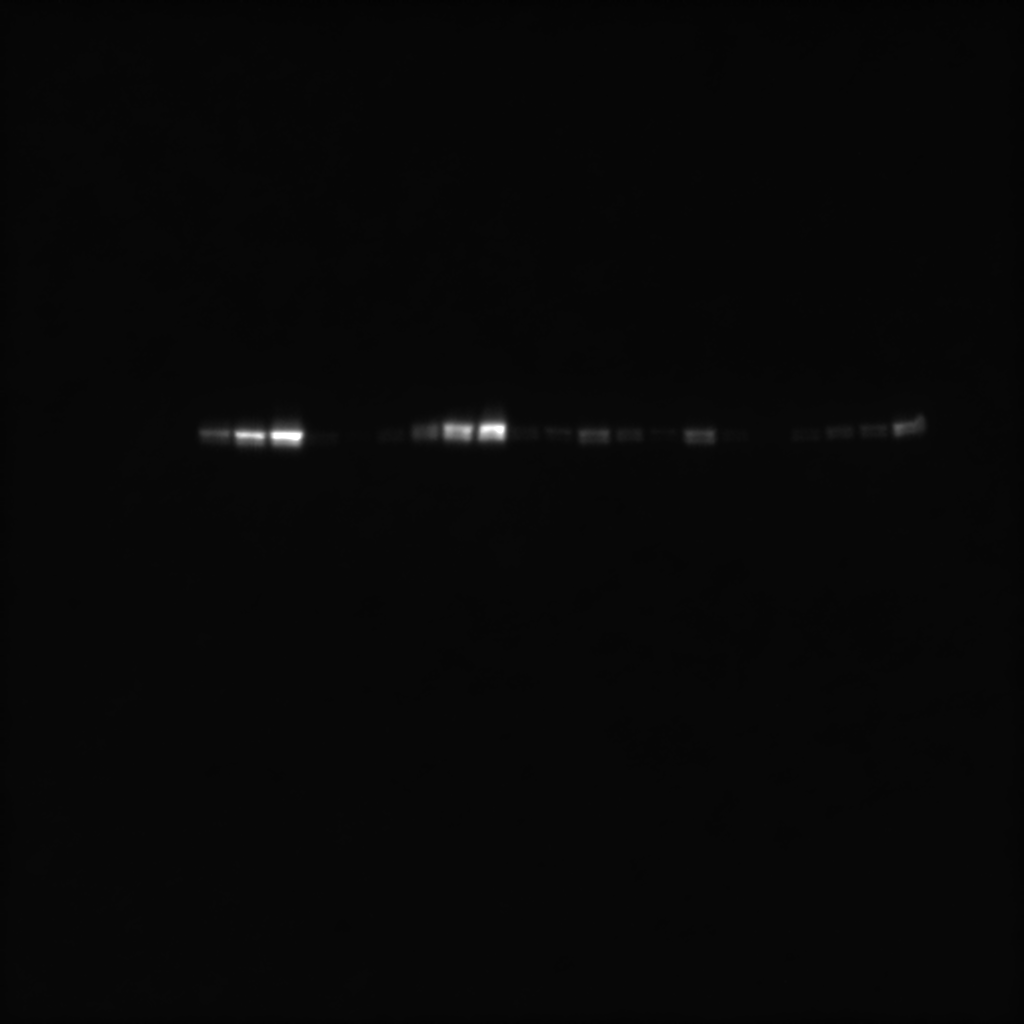

Supplement: Figure 6—source data 1. [file elife-89185-fig6-data1.zip › Figure 6-source data 1/Fig. 6A/IM010751_9Sum (phospho-p62).TIF]

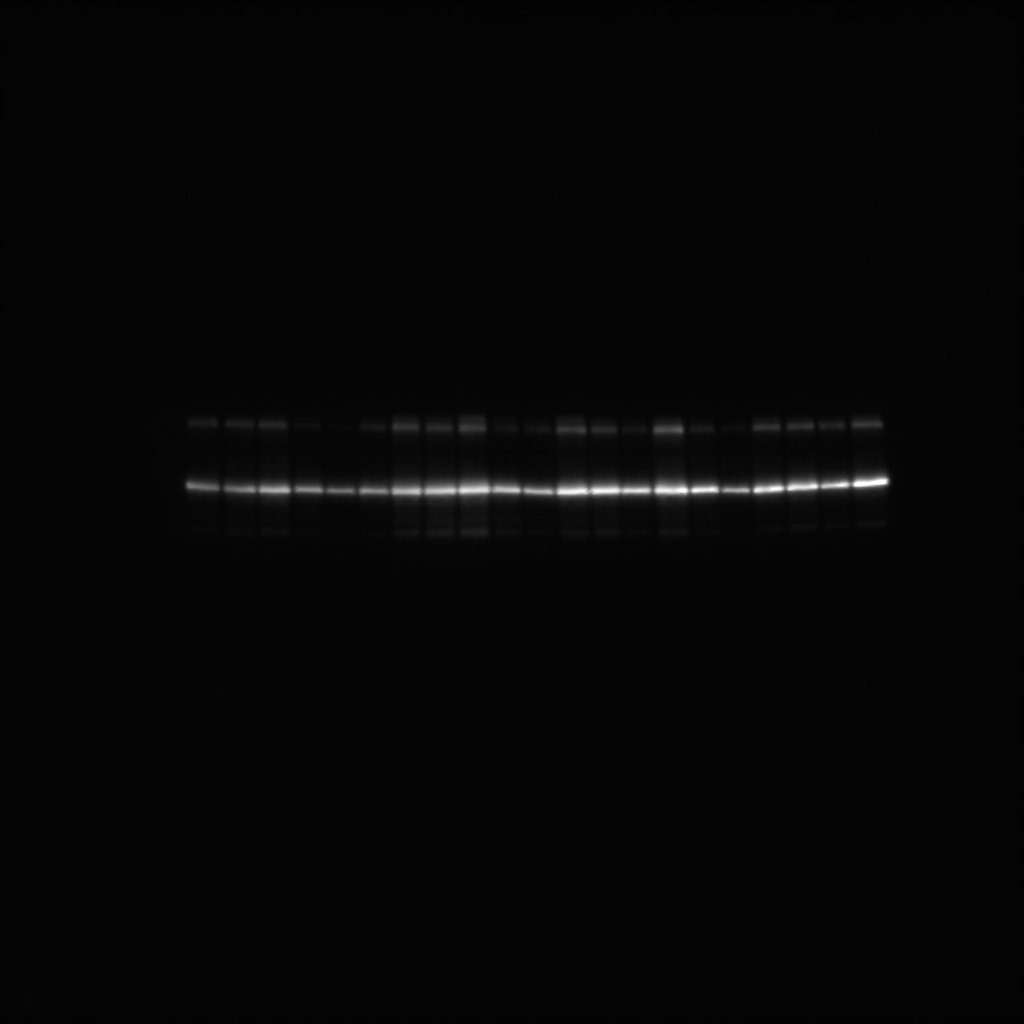

Supplement: Figure 6—source data 1. [file elife-89185-fig6-data1.zip › Figure 6-source data 1/Fig. 6A/IM010756_6Sum (p62).TIF]

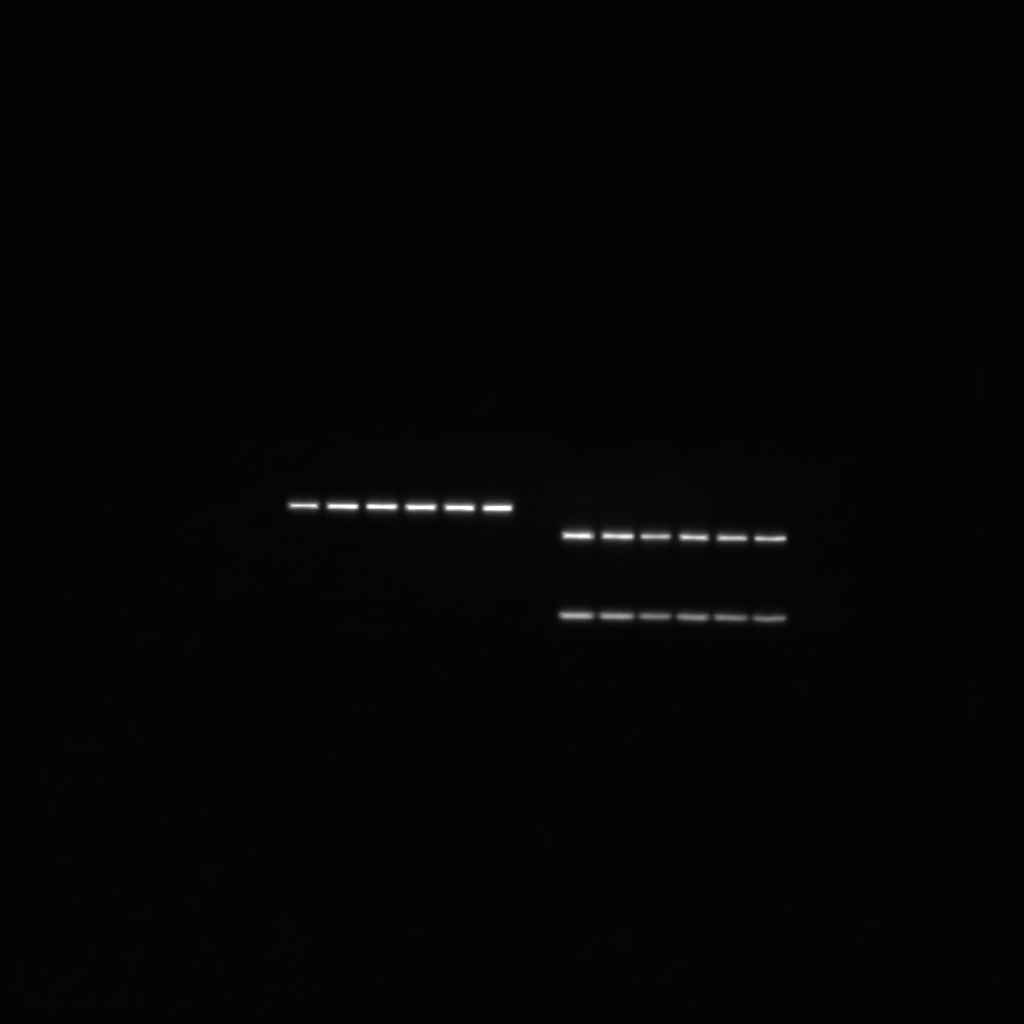

Supplement: Figure 6—source data 1. [file elife-89185-fig6-data1.zip › Figure 6-source data 1/Fig. 6F/IM010833_5Sum (HSP90).TIF]

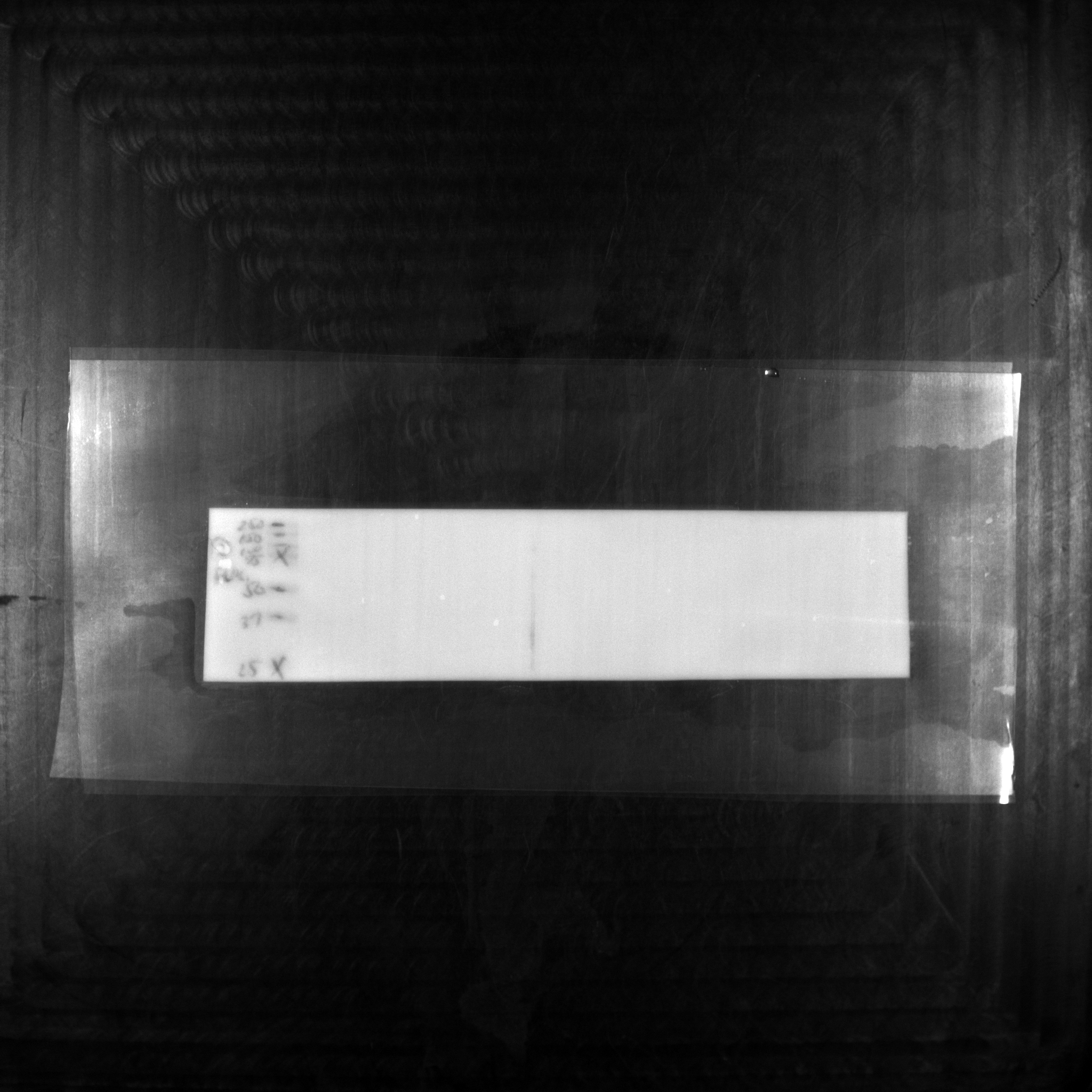

Supplement: Figure 6—source data 1. [file elife-89185-fig6-data1.zip › Figure 6-source data 1/Fig. 6F/IM010857_marker (FLAG).Tif]

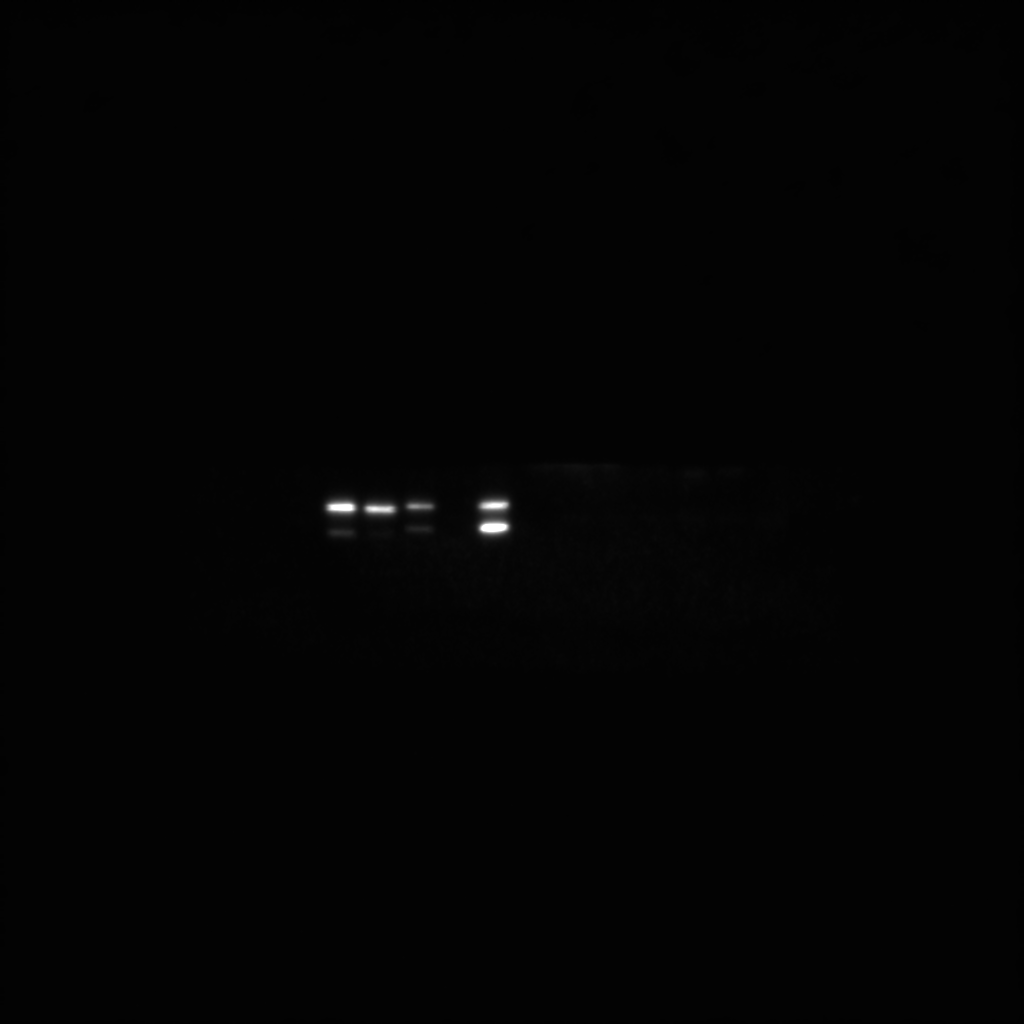

Supplement: Figure 6—source data 1. [file elife-89185-fig6-data1.zip › Figure 6-source data 1/Fig. 6F/IM010853_3Sum (MBL135-3).TIF]

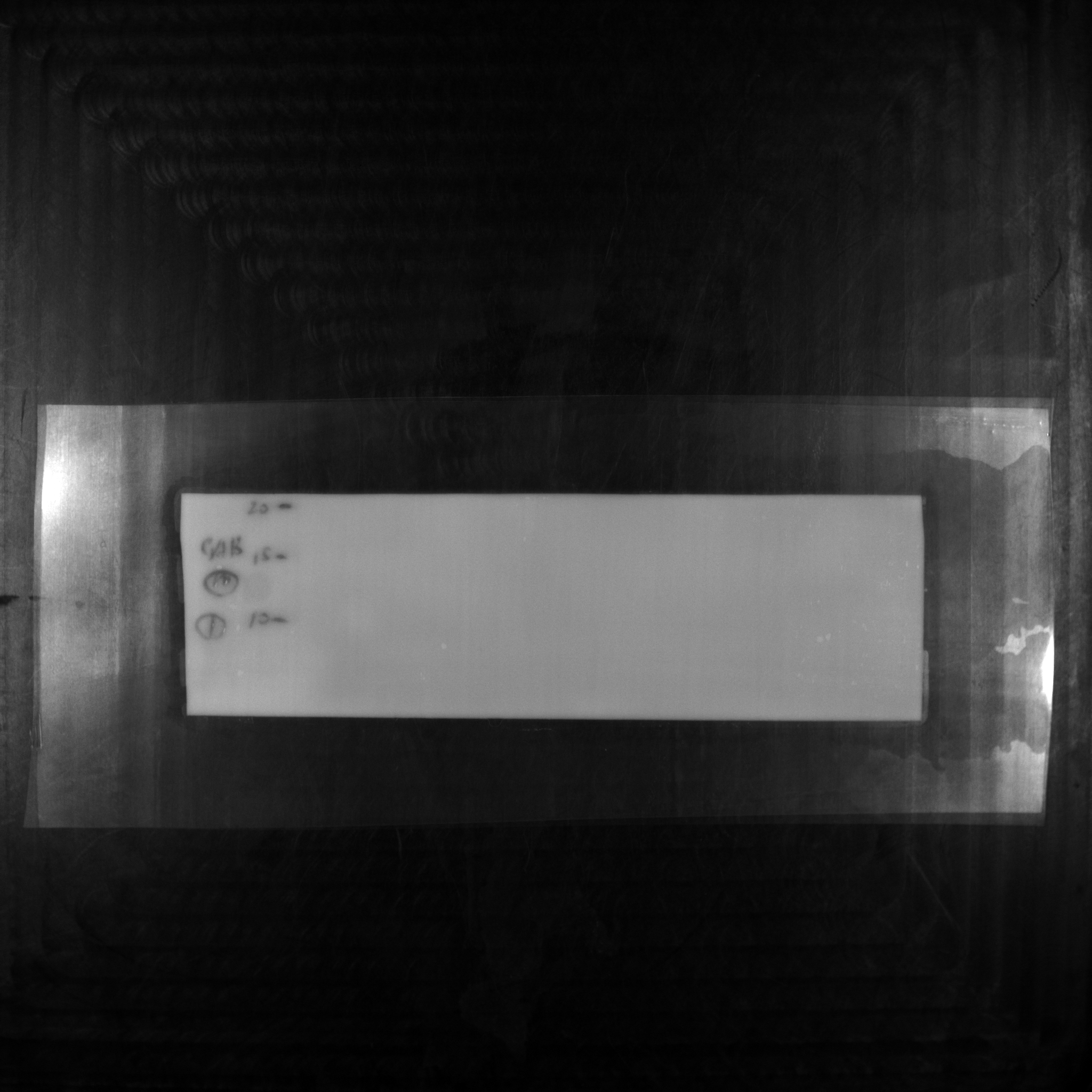

Supplement: Figure 6—source data 1. [file elife-89185-fig6-data1.zip › Figure 6-source data 1/Fig. 6F/IM010850_marker (MBL135-3).Tif]

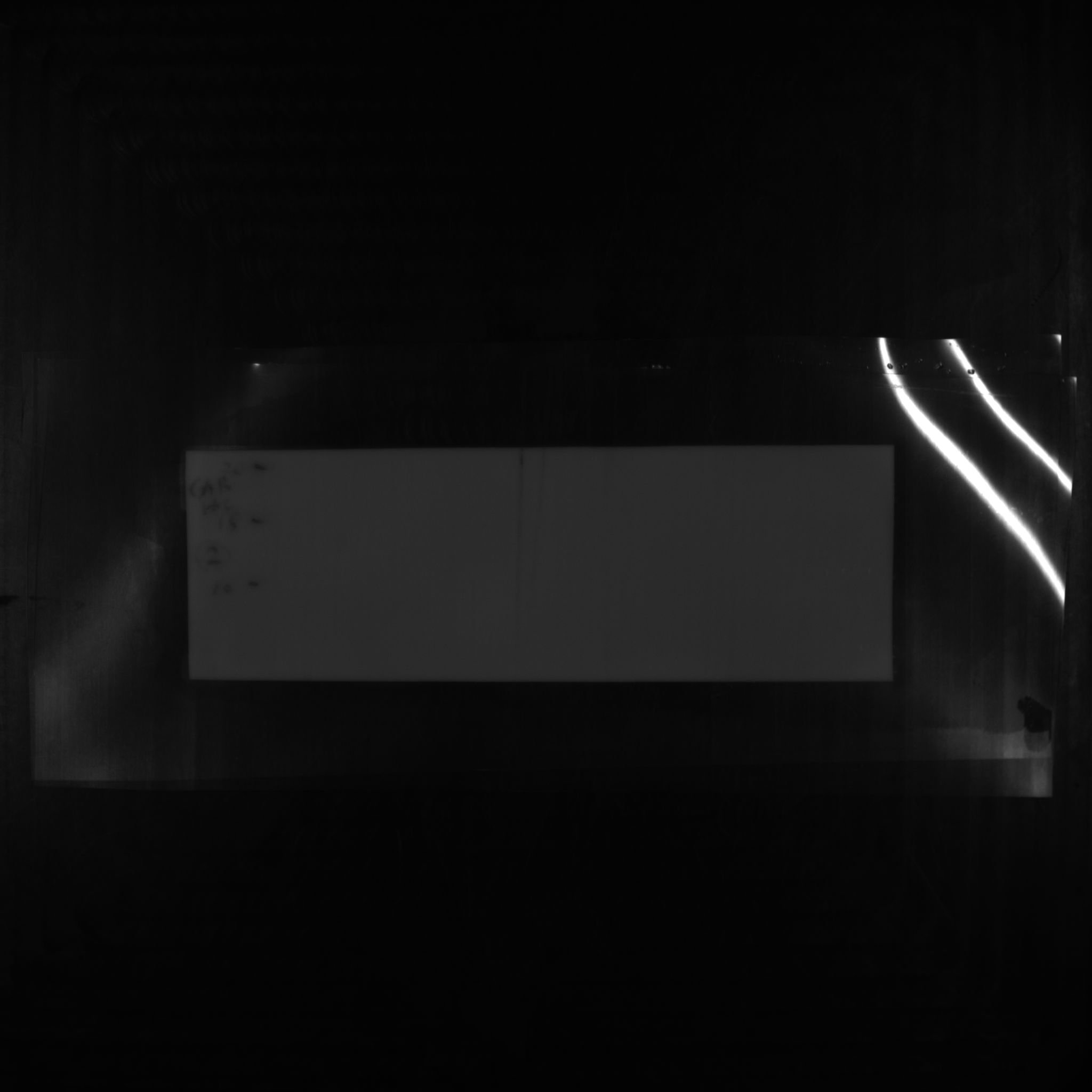

Supplement: Figure 6—source data 1. [file elife-89185-fig6-data1.zip › Figure 6-source data 1/Fig. 6F/IM010854_marker (GABARAP CST13733).Tif]

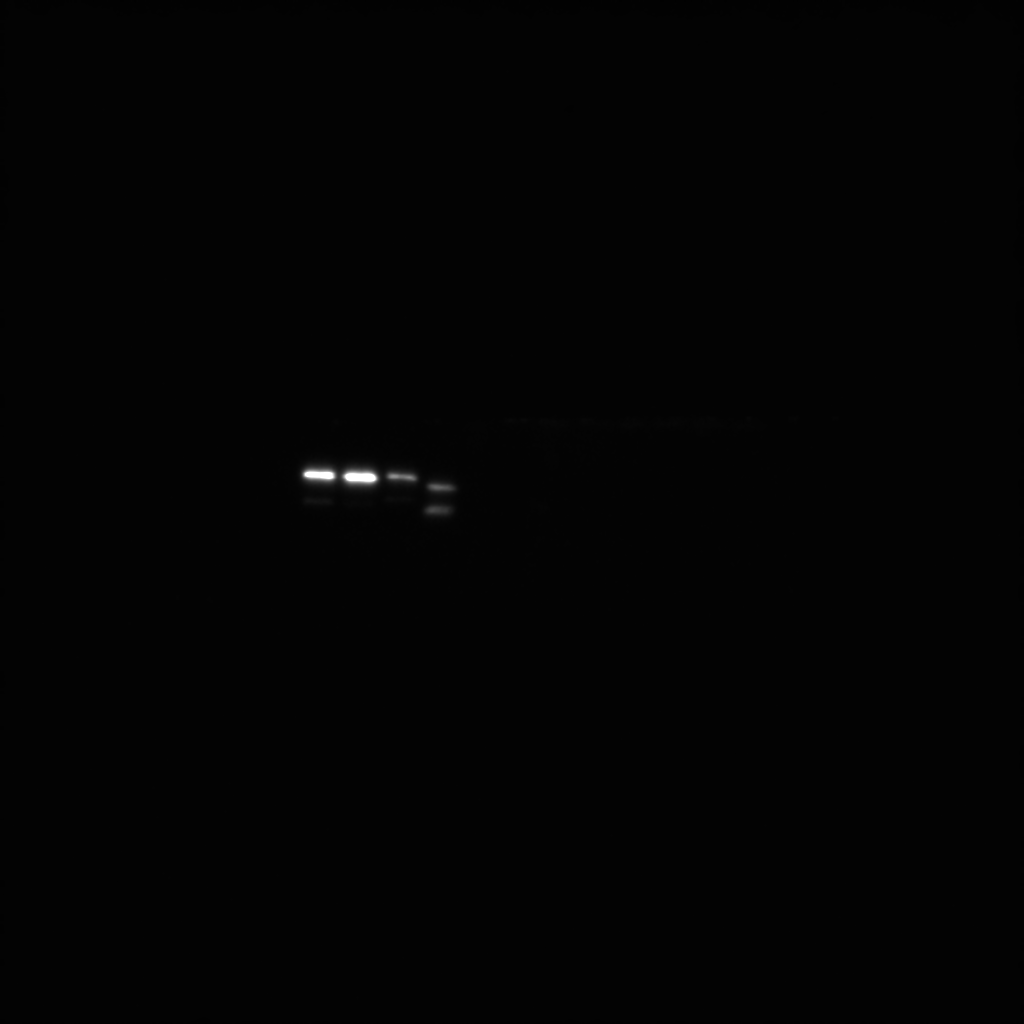

Supplement: Figure 6—source data 1. [file elife-89185-fig6-data1.zip › Figure 6-source data 1/Fig. 6F/IM010856_3Sum (GABARAP CST13733).TIF]

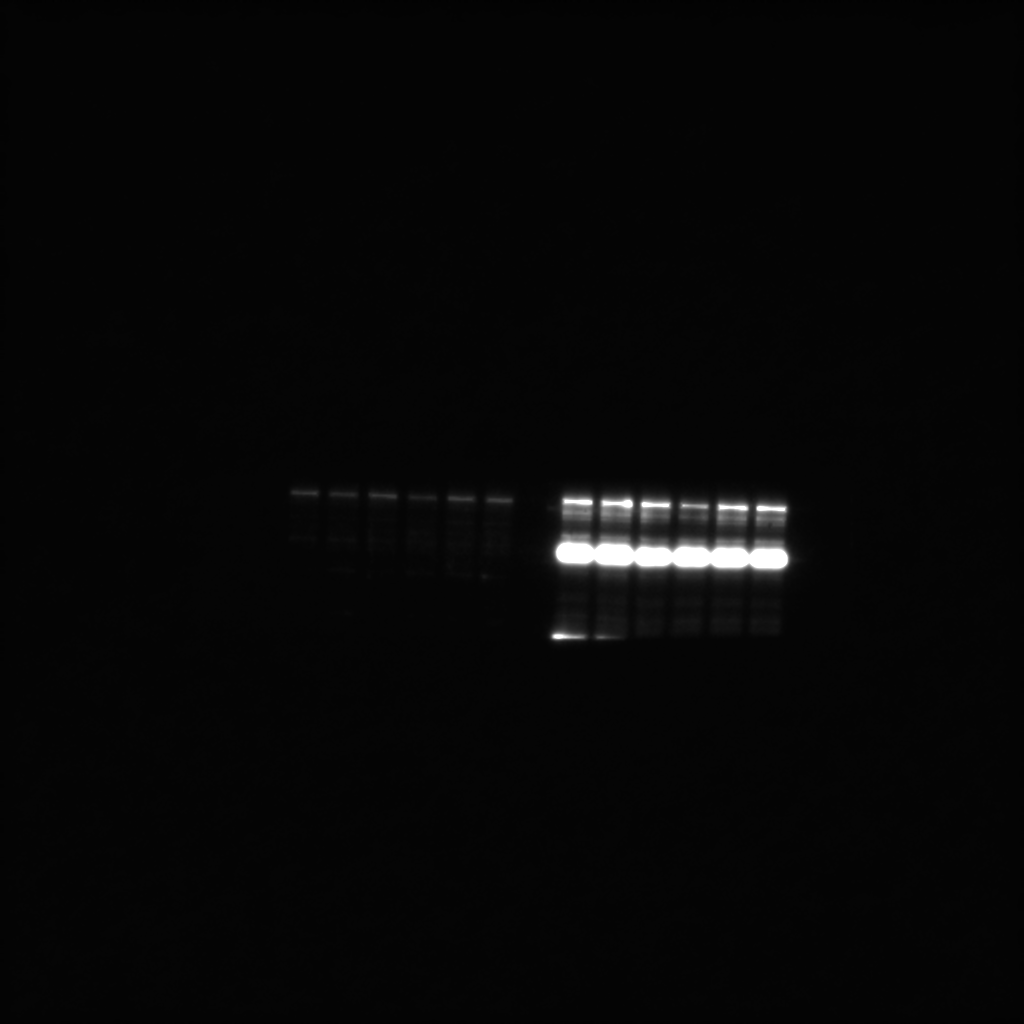

Supplement: Figure 6—source data 1. [file elife-89185-fig6-data1.zip › Figure 6-source data 1/Fig. 6F/IM010858_5Sum (FLAG).TIF]

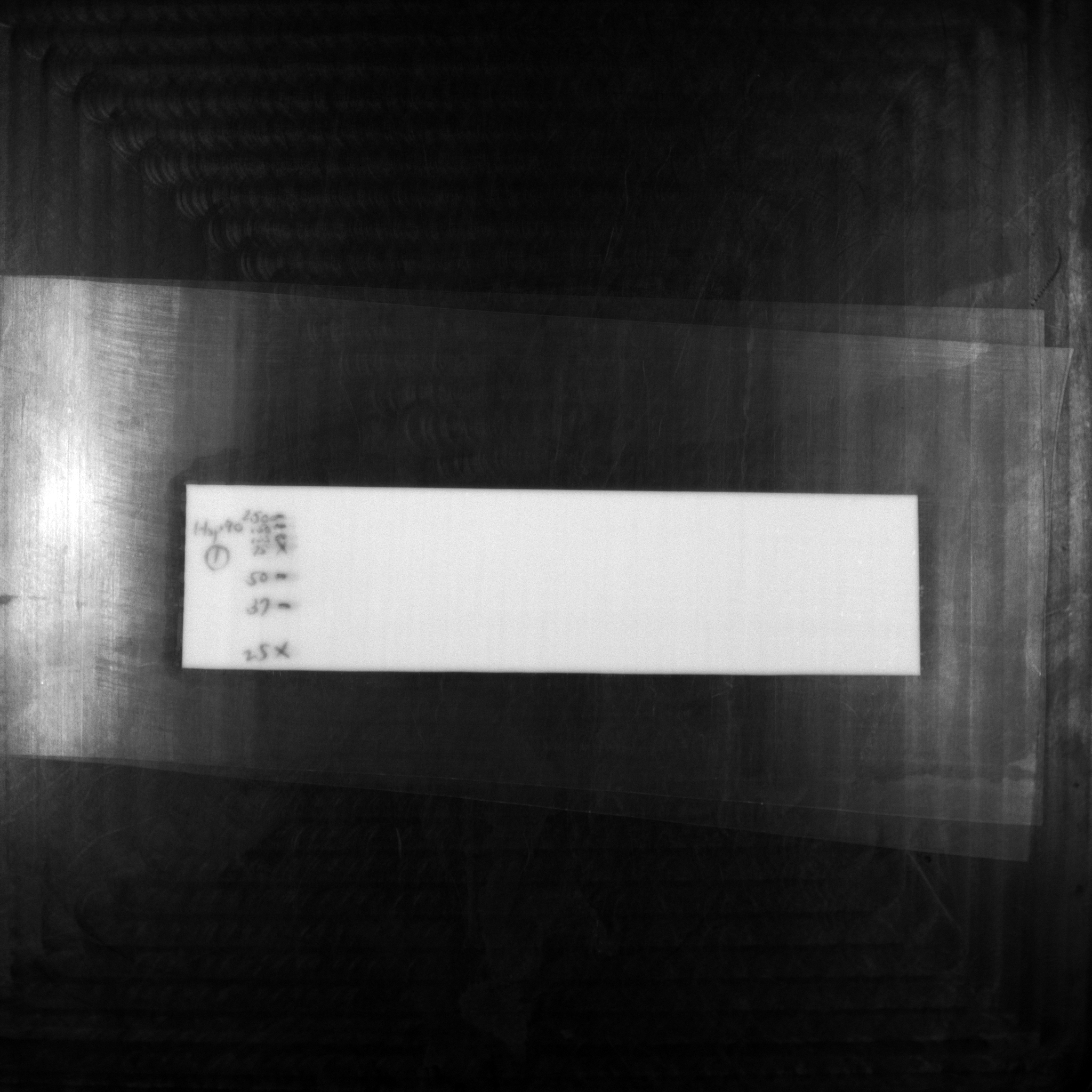

Supplement: Figure 6—source data 1. [file elife-89185-fig6-data1.zip › Figure 6-source data 1/Fig. 6F/IM010832_marker (HSP90).Tif]
