# Supplementary material for: Autophagosome membrane expansion is mediated by the N-terminus and cis-membrane association of human ATG8s
Source: eLife. 2023 Jun 8;12:e89185. doi: 10.7554/eLife.89185 (PMC10289813; doi:10.7554/eLife.89185)
Supplement: Supplementary file 1. [file elife-89185-supp1.docx]

**Table S1. Constructs used in this study**

| REAGENT or RESOURCE | SOURCE | DESCRIPTIONS |
| --- | --- | --- |
| Recombinant DNA | | |
| pAL-GST-LC3B_(1-120aa)_ S3C | This study | Bacterial expression, 3C cleavage |
| pAL-GST-LC3B_(1-120aa)_ S3C K42E/Q43E/L44E | This study |  |
| pAL-GST-LC3B_(1-120aa)_ S3C R68E/R69E/R70E | This study |  |
| pAL-GST-GABARAP_(1-116aa)_ K2C | This study |  |
| pAL-GST-GABARAP_(1-116aa)_ K2C A39E/R40E/I41E | This study |  |
| pAL-GST-GABARAP_(1-116aa)_ K2C R65E/K66E/R67E | This study |  |
| pGEX-6P1-GST-LC3B_(1-120aa)_-His_6_ S3C | This study |  |
| pGEX-6P1-GST-LC3B_(1-120aa)_-His_6_ K42C | This study |  |
| pGEX-6P1-GST-LC3B_(1-120aa)_-His_6_ L44C | This study |  |
| pGEX-6P1-GST-LC3B_(1-120aa)_-His_6_ R69C | This study |  |
| pGEX-6P1-GST-LC3B_(1-120aa)_-His_6_ R70C | This study |  |
| pAL-GST-GABARAP_(1-116aa)_-His_6_ K2C | This study |  |
| pAL-GST-GABARAP_(1-116aa)_-His_6_ V4C | This study |  |
| pAL-GST-GABARAP_(1-116aa)_-His_6_ V4C Nmut (M1E/K2E/E7A) | This study |  |
| pAL-GST-GABARAP_(1-116aa)_-His_6_ A39C | This study |  |
| pAL-GST-GABARAP_(1-116aa)_-His_6_ R40C | This study |  |
| pAL-GST-GABARAP_(1-116aa)_-His_6_ R65C | This study |  |
| pAL-GST-GABARAP_(1-116aa)_-His_6_ K66C | This study |  |
| pAL-GST-GABARAP_(1-116aa)_-His_6_ | This study |  |
| pAL-GST-GABARAP_(10-116aa)_-His_6_ ΔN9 | This study |  |
| pAL-GST-GABARAP_(1-116aa)_-His_6_ Nmut (M1E/K2E/E7A) | This study |  |
| pAL-GST-GABARAP_(1-116aa)_ | This study |  |
| pAL-GST-GABARAP_(10-116aa)_ ΔN9 | This study |  |
| pAL-GST-LC3B_(1-120aa)_ | This study |  |
| pAL-GST-LC3B_(12-120aa)_ ΔN11 | This study |  |
| pGEX-6P1-GST-ATG3 | Landajuela et al., 2016 |  |
| pBacPAK-his_3_-GST-ATG7 | This study | Sf9 insect cell expression, 3C cleavge |
| pFBDM- ATG7-ATG10-ATG12-StrepII^2x^-ATG5-ATG16L1 | This study | High Five insect cell expression, desthiobiotin elution |
| pMRX-ISU-hGABARAP | This study | Retrovirus infection |
| pMRX-ISU-hGABARAP Δ9 | This study |  |
| pMRX-ISU-hGABARAP ARI-EEE | This study |  |
| pMRX-ISU-hGABARAP Nmut(M1E/K2E/E7A) | This study |  |
| pMRX-ISU-hGABARAP Y49A L50A | This study |  |
| pMRX-IPU-EGFP-STX17TM | Tsuboyama et al., 2016 | Retrovirus infection |
| pQCXIH-ATG2A-FLAG | Tamura et al., 2017 | Expression in mammalian cells |

LANDAJUELA, A., HERVAS, J. H., ANTON, Z., MONTES, L. R., GIL, D., VALLE, M., RODRIGUEZ, J. F., GONI, F. M. & ALONSO, A. 2016. Lipid Geometry and Bilayer Curvature Modulate LC3/GABARAP-Mediated Model Autophagosomal Elongation. *Biophys J,* 110**,** 411-422.

TAMURA, N., NISHIMURA, T., SAKAMAKI, Y., KOYAMA-HONDA, I., YAMAMOTO, H. & MIZUSHIMA, N. 2017. Differential requirement for ATG2A domains for localization to autophagic membranes and lipid droplets. *FEBS Lett,* 591**,** 3819-3830.

TSUBOYAMA, K., KOYAMA-HONDA, I., SAKAMAKI, Y., KOIKE, M., MORISHITA, H. & MIZUSHIMA, N. 2016. The ATG conjugation systems are important for degradation of the inner autophagosomal membrane. *Science,* 354**,** 1036-1041.
